# Combined Analysis of the Metabolome and Transcriptome Identified Candidate Genes Involved in Phenolic Acid Biosynthesis in the Leaves of *Cyclocarya paliurus*

**DOI:** 10.3390/ijms21041337

**Published:** 2020-02-17

**Authors:** Weida Lin, Yueling Li, Qiuwei Lu, Hongfei Lu, Junmin Li

**Affiliations:** 1 College of Life Science, Zhejiang Sci-Tech University, Hangzhou 310018, China; 15267665350@163.com (W.L.); luhongfei0164@163.com (H.L.); 2 Zhejiang Provincial Key Laboratory of Plant Evolutionary Ecology and Conservation, Taizhou University, Taizhou 318000, China; liyl@tzc.edu.cn (Y.L.); luqiuwei2020@163.com (Q.L.)

**Keywords:** phenolic acid, *Cyclocarya paliurus*, metabolome, transcriptome, developmental stages

## Abstract

To assess changes of metabolite content and regulation mechanism of the phenolic acid biosynthesis pathway at different developmental stages of leaves, this study performed a combined metabolome and transcriptome analysis of *Cyclocarya paliurus* leaves at different developmental stages. Metabolite and transcript profiling were conducted by ultra-performance liquid chromatography quadrupole time-of-flight tandem mass spectrometer and high-throughput RNA sequencing, respectively. Transcriptome identification showed that 58 genes were involved in the biosynthesis of phenolic acid. Among them, 10 differentially expressed genes were detected between every two developmental stages. Identification and quantification of metabolites indicated that 14 metabolites were located in the phenolic acid biosynthetic pathway. Among them, eight differentially accumulated metabolites were detected between every two developmental stages. Association analysis between metabolome and transcriptome showed that six differentially expressed structural genes were significantly positively correlated with metabolite accumulation and showed similar expression trends. A total of 128 transcription factors were identified that may be involved in the regulation of phenolic acid biosynthesis; these include 12 MYBs and 10 basic helix–loop–helix (bHLH) transcription factors. A regulatory network of the phenolic acid biosynthesis was established to visualize differentially expressed candidate genes that are involved in the accumulation of metabolites with significant differences. The results of this study contribute to the further understanding of phenolic acid biosynthesis during the development of leaves of *C. paliurus*.

## 1. Introduction

Natural phenolic compounds can be commonly found in plants and form the largest group of phytochemicals. Polyphenols are secondary metabolites of plants and contribute to the bitterness, astringency, color, flavor, odor in food [1]. Polyphenols can be divided into different groups, such as phenolic acids, flavonoids, tannins, stilbenes and lignans [1]. These compounds have different roles, such as signaling molecules, plant defense agents and auxin transport regulators [2]. In addition, these compounds have been well investigated due to their antioxidant activity and ability to scavenge free radicals. They also have various health benefits such as anti-inflammatory, antibacterial, anti-proliferative, anti-cancer, anti-oxidant properties [3,4,5].

Phenolic acids, the hydroxyl derivatives of aromatic carboxylic acids, are widely distributed in plants and have been classified into two categories: hydroxybenzoic acid derivatives and hydroxy cinnamic acid derivatives [6]. Phenolic acids are produced in plants via the phenylpropanoid pathway, using shikimic acid. These are by-products of the monolignol pathway and as breakdown products of lignin and cell wall polymers in vascular plants [7]. Two main amino acids that are involved in the synthesis of phenolic acids in plants include phenylalanine and, to a lesser extent, tyrosine [8,9]. Deamination, hydroxylation and methylation are the three main reactions that are involved in the formation of phenolic acids [10]. Firstly, a deamination of the phenylalanine and/or the tyrosine occurs, yielding cinnamic and/or p-coumaric acids, respectively. The aromatic rings of cinnamic and p-coumaric acid are then hydroxylated and methylated to form its derivatives ferulic and caffeic acids [11]. Moreover, these phenolic acids contain C_6_–C_3_ structures and are used as precursors in the synthesis of lignins and other phenolics [12]. Less knowledge about phenolic acid biosynthesis is available than about flavonoids and monolignols and many steps of the pathway remain unknown to date [13].

Recently, phenolic acids received increasing attention; however, most studies of plant phenolics focused on examining these compounds in vegetables and fruits [14,15]. Mehrabani and Hassanpouraghdam showed that the total phenolic acids content was highest in the fruits during the fruit early growing stage in both “Zonouz” and “Gala” apples [16]. Furthermore, chlorogenic acid was the principal phenolic acid component during the early stage of fruit growth, while caffeic acid had the highest level at harvest time. Both the concentrations and types of phenolic compounds were high in young loquat fruit but decreased steadily during growth. However, the concentration of chlorogenic acid increased during ripening and became predominant in ripe fruit [17]. Payyavula et al. reported that the major phenolic—5-chlorogenic acid (5CGA)—decreased during potato development and the expression of most phenylpropanoid structural genes decreased during their development [18]. Ma et al. found that nine phenolic acid biosynthesis pathway genes exhibited three distinct expression patterns during wheat filling, suggested that the expression of phenolic acid biosynthesis genes may be closely related to the accumulation of phenolic acids [2].

Metabolomics data can provide a wealth of information on the biochemical status of tissues, the interpretation of which offers an effective approach that can be used for the functional characterization of genes [19,20]. Furthermore, metabolomics analyses can be regarded as a technical means for an association analysis. In combination with other data, this can be used to analyze the gene function involved in the metabolic pathway of interest and can also provide supporting information for gene mining [21]. The recent development and application of high-throughput sequencing, high-resolution mass spectrometry and information processing technologies has become indispensable to systems biology research for the exploration of major biological phenomena [22,23]. Transcript and metabolite datasets have been combined through correlation and clustering analyses and can be represented as connection networks between genes and metabolites [24], which can reveal the response mechanism of rice to elevated night temperature [25], the regulation mechanism of delphinidin in flower color in grape hyacinth [26], the potato pigmentation mechanism [27], the blue flower formation mechanism in waterlily [28] and catechin production in albino tea cultivar “YuJin-Xiang” [29]. Furthermore, the integration of transcriptomics and metabolomics offers notable advantages to identify the biosynthetic mechanisms of key metabolic pathways [30,31]. Until now, the biosynthesis of phenolic compounds has not been investigated by the combined analysis of transcriptomics and metabolomics data.

*Cyclocarya paliurus* (Batal) Iljinskaja, the sole species of the genus *Cyclocarya*, is commonly known as the “sweet tea tree” in China and is used as a traditional Chinese medicine. It is widely distributed throughout the sub-tropical regions of China [32,33]. In China, *C. paliurus* has been traditionally used as a health food for more than 1000 years [34]. The leaves of *C. paliurus* have been used as functional food or as nutraceutical tea for the treatment of hyperhidrosis, hypertension and diabetes mellitus [35,36]. In 2013, *C. paliurus* leaves have been approved as a new food raw material by the National Health and Family Planning Commission of China [37]. It has been suggested that the biological active substances in *C. paliurus* leaves are responsible for its therapeutic effects [38]. Due to its various health benefits, a large production of *C. paliurus* leaves is required [39]. The accumulation of phenolic acids and the expression patterns of phenolic acid biosynthesis genes in the leaves of *C. paliurus* have not been investigated at different developmental stages. In this study, integrated transcriptomics and metabolomics techniques were used to investigate the changes in phenolic acid content and the different expressed gene involved in the synthesis pathway of phenolic acid, we aimed to find out that: 1) the phenolic acid synthesis pathway of *C. paliurus* leaves; 2) the regulatory mechanisms underlying the synthesis of phenolic acid in the leaves of *C. paliurus* at different developmental stages. The results of the present study add information on the accumulative dynamics of phenolic compounds in the leaves of *C. paliurus* at different developmental stages and provide a valuable reference for determining time for the harvest of leaves.

## 2. Results

### 2.1. Expression of Phenolic Acid Biosynthesis Genes in Leaves of C. Paliurus at Different Developmental Stages

**Figure 1:**
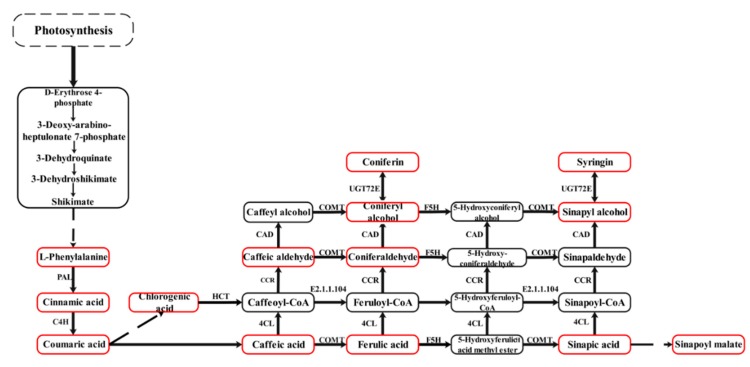
The phenolic acid synthesis pathway of *Cyclocarya paliurus* leaves. The red squares indicate detected metabolites and the dashed lines indicate a series of enzymatic reactions. PAL, phenylalanine ammonia-lyase; C4H, trans-cinnamate 4-monooxygenase; F5H, ferulate-5-hydroxylase; COMT, caffeic acid 3-O-methyltransferase; CAD, cinnamyl-alcohol dehydrogenase; UGT72E, coniferyl-alcohol glucosyltransferase; HCT, shikimate O-hydroxycinnamoyltransferase; EC: 2.1.1.104, caffeoyl-CoA O-methyltransferase; 4CL, 4-coumarate--CoA ligase; CCR, cinnamoyl-CoA reductase.

**Figure 2:**
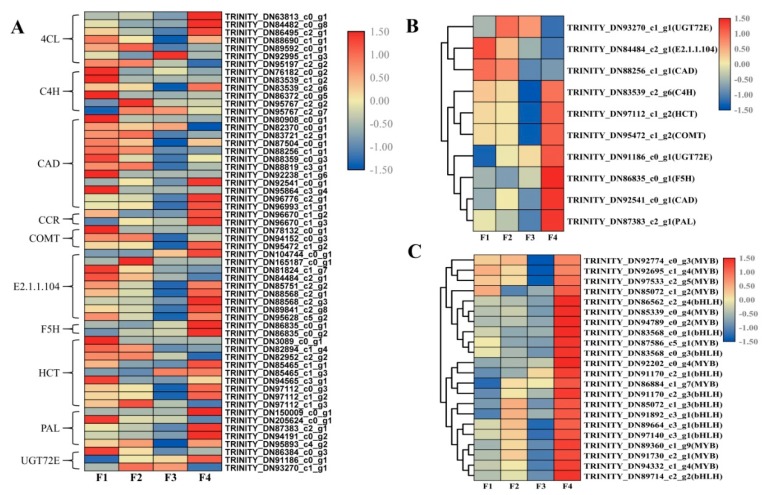
The heat map of phenolic acid biosynthesis pathway genes (**A**), the differential gene expression in phenolic acid biosynthesis pathway (**B**) and the heat map of MYB and bHLH transcription factors genes (**C**).

The phenolic acid synthesis pathway of *C. paliurus* leaves was constructed based on the detected metabolites in reference to the phenylpropanoid biosynthesis in the KEGG database (Figure 1). A total of 58 genes were identified to be involved in the phenolic acid biosynthesis pathway (Figure 2A). Comparison of the four stages of development identified a total of 10 DEGs, including one PAL gene (TRINITY_DN87383_c2_g1), one C4H gene (TRINITY_DN83539_c2_g6), one F5H gene (TRINITY_DN86835_c0_g1), one COMT gene (TRINITY_DN95472_c1_g2), two CAD genes (TRINITY_DN88256_c1_g1, TRINITY_DN92541_c0_g1), two UGT72E genes (TRINITY_DN91186_ c0_g1, TRINITY_DN93270_c1_g1), one HCT gene (TRINITY_DN97112_c1_g2) and one caffeoyl- CoA O-methyltransferase gene (TRINITY_DN84484_c2_g1) (Figure 2B). The expressions of two DEGs (TRINITY_DN84484_c2_g1, TRINITY_DN88256_c1_g1) decreased with progressing developmental stages and the gene expression was highest during the F1 stage. The expression of only one DEG (TRINITY_DN93270_c1_g1) increased at first and then decreased and was highest during the F2 stage. The other DEGs followed different expression trends but all of their gene expression levels were highest during the F4 stage. A total of 22 transcription factors (TFs), including 12 MYB TFs and 10 basic helix–loop–helix (bHLH) TFs, were involved in the phenolic acid biosynthesis pathway (Figure 2C).

### 2.2. Identified Metabolites Involved in Phenolic Acid Biosynthesis Pathway

A total of 14 metabolites involved in the phenolic acid biosynthetic pathway were detected, including six phenolic acids (cinnamic acid, caffeic acid, chlorogenic acid, ferulic acid, sinapic acid and p-coumaric acid) (Table 1). Differentially accumulated metabolites (DAMs) were defined as those that exhibit a fold change ≥ 2 or a fold change ≤ 0.5 and a variable importance in project (VIP) ≥ 1 between the stages of leaf development. Eight DAMs were detected, including two phenolic acids (p-coumaric acid, sinapic acid) and other metabolites were identified as syringin, coniferin, sinapyl alcohol, sinapoyl malate, L-phenylalanine and coniferyl alcohol. The DAMs contents showed different expression trends during the four developmental stages but all the eight metabolite contents were highest at the F4 stage.

### 2.3. Comprehensive Analysis of Metabolome and Transcriptome

**Figure 3:**
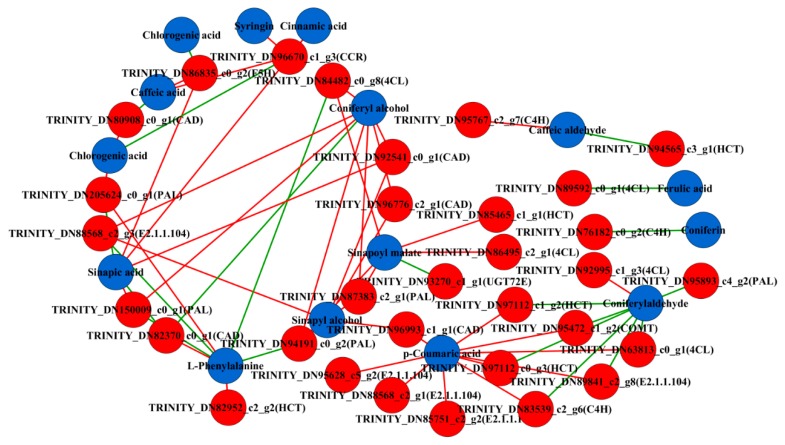
Co-expression analysis of structural genes and metabolites of phenolic acid biosynthesis pathway in the leaves of *Cyclocarya paliurus* at different developmental stages. Blue nodes represent metabolites and red nodes represent genes. Red edges represent positive correlations and green edges represent negative correlations.

To clearly determine the association between the genes and metabolites, the transcriptome and the metabolite data were integrated by correlation analysis and networks were constructed to determine transcript-metabolite correlations. The metabolite and transcriptome data were log_2_-transformed prior to correlation analysis. Only correlation pairs with a Pearson correlation coefficient (PCC) ≥ 0.9 and a *p* value ≤ 0.05 were included in the analysis. A total of 64 related pairs were identified and visualized using Cytoscape software (version 3.6.1) (Supplementary material 1). The visualized network showed that a total of 47 nodes were connected by 64 edges. These 47 nodes include 32 genes and 15 metabolites. Furthermore, 43 pairs had a positive correlation and 21 pairs were negatively correlated (Figure 3). In the phenolic acid biosynthesis, a total of six candidate DEGs were found, including *PAL* (TRINITY_DN87383_c2_g1), *C4H* (TRINITY_DN83539_c2_g6), *CAD* (TRINITY_DN92541_c0_g1), *COMT* (TRINITY_DN95472_c1_g2), *HCT* (TRINITY_DN97112_c1_g2) and *UGT72E* (TRINITY_DN93270_c1_g1). These results indicated that several DEGs were highly correlated with the corresponding metabolites that are involved in phenolic acid biosynthesis. The transcriptome data validated the authenticity and accuracy of the metabolic analysis.

### 2.4. Screening of Transcription Factors Related to Phenolic Acid Biosynthesis and Phylogenetic Analysis

**Figure 4:**
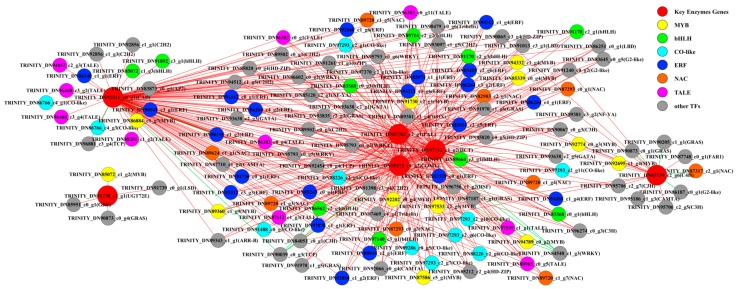
Co-expression analysis of structural genes of phenolic acid biosynthesis pathway and transcription factors (TFs) in the leaves of Cyclocarya paliurus. Red nodes represent genes. Yellow nodes represent MYB TFs. Green nodes represent bHLH TFs. Bright blue nodes represent CO-like TFs. Blue nodes represent ethylene responsive transcription factors (ERF). Pink nodes represent transcription activator-like effector (TALE) TFs. Orange nodes represent NAC (NAM, ATAF, and CUC) TFs. Grey nodes represent the other TFs. Red edges represent positive correlations and green edges represent negative correlations.

To systemically identify unknown putative transcription factors that control the phenolic acid biosynthesis of *C. paliurus* during the different developmental stages, co-expression analysis was employed between metabolic pathway genes and differentially expressed transcription factor (TF) genes. Six phenolic acid synthesis genes were previously screened (genes encoding PAL, C4H, CAD, COMT, UGT72E and HCT) and were selected as “guiding genes” to identify co-expression specific relationships. A total of 386 differentially expressed TFs were identified via comparison with the PlantTFDB database. Correlation analysis was performed between guiding genes and differentially expressed TFs and correlation pairs with PCC ≥ 0.9 and *p*-value ≤ 0.05 were selected. A total of 414 related pairs were identified and visualized using Cytoscape software (version 3.6.1) (Supplementary material 2). The visualized network showed that a total of 134 nodes were connected by 414 edges and these 134 nodes included six guiding genes and 128 TFs (Figure 4). The members of MYB, BHLH and ERF families were the most abundant positive correlation TFs among the identified co-expressed TFs (*p*-value ≤ 0.05).

Since MYB and bHLH are two of the most important transcription factor families that are involved in the biosynthesis of phenolic acid [23,40], the MYB and bHLH gene of *C. paliurus* was selected to construct a phylogenetic tree with *Arabidopsis* R2R3MYB TFs and *Arabidopsis* bHLH TFs, respectively. 12 MYB TFs could be divided into six clusters, where the TRINITY_DN86884_c1_g7, TRINITY_DN92774_c0_g3, TRINITY_DN89360_c1_g9, TRINITY_DN91730_c2_g1 and TRINITY_ DN97533_c2_g5 genes showed homology with AtMYB39, AtCDC5 and AtMYB91 and were clustered into a biggest cluster (Supplementary material 3). The other MYB genes were divided into another five groups (Supplementary material 3). Ten bHLH TFs could be divided into eight clusters, where TRINITY_DN97140_c3_g1 and TRINITY_DN85072_c1_g3 showed homology AtbHLH001 and AtbHLH002 and were clustered into one cluster (Supplementary material 4).

### 2.5. Regulatory Network of Phenolic Acid Biosynthesis

**Figure 5:**
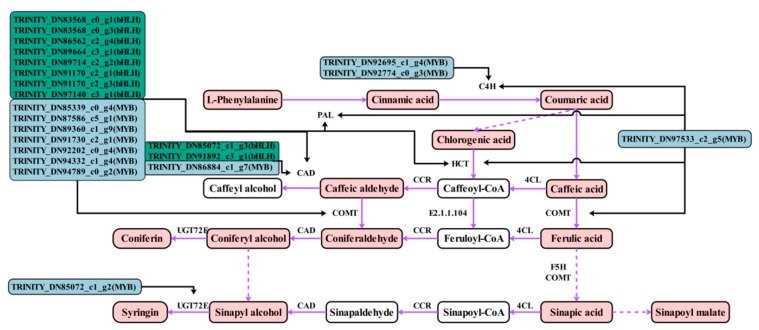
Regulatory network of phenolic acid biosynthesis in the leaves of *Cyclocarya paliurus* at different developmental stages. The red squares indicate detected metabolites. The solid frame arrow represents only one step of process and the dotted frame arrow represents more than one step of process. Black arrows indicate possible directions for MYB and bHLH transcription factors. Purple arrows indicate directions for phenolic acid biosynthesis.

The phenolic acid synthesis pathway of *C. paliurus* leaves was constructed in reference to the phenylpropane biosynthesis method in the KEGG database (Figure 5). The network was established by combining the results of all metabolites, genes and TFs to more directly show the relationship between TF-regulated gene expression, gene expression and metabolite accumulation. Transcription factor bind to the promoter of structural genes. Therefore, we hypothesized that the TFs activate the expression of genes of by binding to the promoters of these structural genes, inducing the accumulation of the metabolites described above. The structural genes of the phenolic acid biosynthesis pathway are regulated by many MYB and bHLH TFs, including PAL, C4H, CAD, COMT and HCT genes. All MYB and bHLH TFs, screened by correlation, showed similar expression trends and were highly expressed during the F4 stage, which was similar to the trend of most structural DEGs (Figure 2C). Specifically, 12 MYB and 10 bHLH TFs and 10 structural DEGs were candidate genes obtained by this study. These can be used for investigating the regulatory mechanism underlying phenolic acid biosynthesis in the leaves of *C. paliurus* at different developmental stages.

### 2.6. RT-qPCR Validation of the Transcriptomic Data

**Figure 6:**
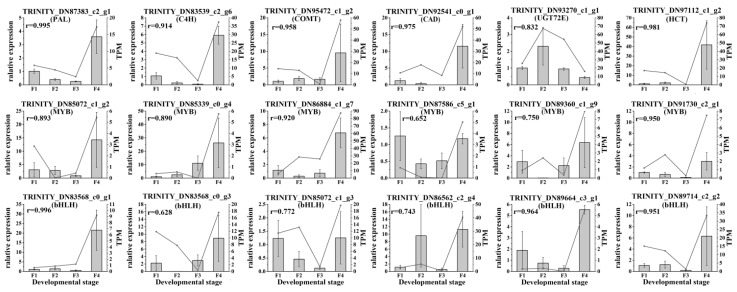
Real-time polymerase chain reaction (PCR) validation of candidate unigenes involved in *Cyclocarya paliurus* phenolic acid biosynthesis by RNA-seq. The histogram shows the relative gene expression obtained via real-time PCR. The transcripts per million (TPM) of each million mapped fragments of the transcriptome are represented with a line graph. The right *y*-axis indicates gene expression levels calculated as TPM. The left *y*-axis indicates relative gene expression levels obtained via real time PCR.

The abundance of gene transcripts of enzymes and TFs related to the *C. paliurus* phenolic acids biosynthesis were determined via qRT-PCR analysis. Based on the differences in expression levels of genes at the four different developmental stages, 18 genes were screened for qRT-PCR, including six structural genes (PAL, C4H, HCT, CAD, COMT and UGT72E), six MYB TFs and six bHLH TFs. All of these 18 selected genes had similar expression patterns than identified in the RNA-seq data (Figure 6). Therefore, the data obtained in this study can be used to study the phenolic acids biosynthesis and metabolism-related genes in *C. paliurus*.

## 3. Discussion

Phenolic acids in plants are primarily derived via the phenylpropanoid biosynthetic pathway [2]. PAL, C4H, 4CL, F5H and COMT are major enzymes in the phenolic acids biosynthesis pathway, which action leads to the production of different phenolic acid compounds. CAD, CCR and UGT72E enzymes mediate the synthesis of different phenolic acid derivatives. HCT, 4CL and E2.1.1.104 lead to the production of different phenolic acid compounds. In this study, all the 10 genes were found differentially expressed at four developmental stages of *C. paliurus* leaves (Figure 2B). However, the expression trends of the 10 genes during four different leaf developmental stages were different. For example, expression of C4H, HCT, COMT and PAL were highest during F4 stage, followed by F1 and F2 stages and lowest during F3 stage; while expression of F5H was highest during F4 stage, followed by F3 and F1 stages and lowest during F2 stage. These genes may play important roles in the accumulation of all examined phenolic acids. It is well known that phenylpropanoid biosynthesis is a very complex process and that multiple enzyme metabolites are produced; moreover, several enzymes exist in many isoforms [41]. Gayoso et al. reported that of six PAL genes examined in tomato roots, PAL2 was the most highly expressed, followed by PAL3, PAL4 and PAL6 [42]. Other reports identified two *Bn*C4H genes in rapeseed and two *Cs*4CL genes in tea [43,44]. This study also showed that multiple genes encode the same enzyme and have different expression patterns. For example, five PAL genes and seven 4CL genes were found in *C. paliurus*. These findings suggest that different genes of the multiple gene family play different roles in the phenolic acid biosynthetic pathway. Further research is required to determine the role these genes play during the biosynthesis of specific phenolic acids.

Metabolomics is an emerging omics technology that, similar to genomics and proteomics, can be used to qualify and quantify metabolites of small molecular weight within the cells of an organism [45]. Plant metabolomics, a new field in the post-genome era [46], has been widely applied for the investigation of patterns of metabolite accumulation. Furthermore, this technology was used to investigate the underlying genetic basis of these patters by identifying genes involved in the metabolism, which is currently a topic of interest in modern plant biology. Metabolites are the final products of cell biological regulation process and their levels can be regarded as the response of plant development to genetic and environmental changes [47]. This study performed metabolomics analysis of *C. paliurus* leaves at different developmental stages and identified a total of 14 metabolites of phenolic acid biosynthetic pathway, eight of which were differentially accumulated. L-phenylalanine, as a precursor of the phenolic acid biosynthesis pathway, accumulated during the first three stages and reached a peak during F3 stage, while decreased significantly during the F4 stage (Table 1). The other differentially expressed metabolites also showed different accumulation trend during the four developmental stages, they were actually highest during the F4 stage. These results indicated that phenolic acids and their derivatives accumulate in leaf during the development. Non-differentially expressed metabolites may be affected by upstream or downstream pathways and do not show similar expression trends.

Since transcriptome analysis is regarded as a central way to study the expression level, structure and function of genes to identify phenotypic traits, the combined analysis of both transcriptome and metabolome has increasingly become a popular and practical tool for the mining of new genes that may be involved in various metabolic pathways [48]. Thus, this method will likely become an effective tool that can be used to investigate the mechanisms involved in the regulation of the phenolic acid biosynthesis in *C. paliurus*. In this study, metabonomic data and transcriptome profiling analysis were conducted in combined analysis to identify genes involved in phenolic acid biosynthesis. This enabled the search for useful information about the accumulation and regulation of phenolic acids in the leaves of *C. paliurus* during different developmental stages. Six DEGs that were significantly associated with metabolites were screened by correlation network analysis (PAL, C4H, COMT, HCT, CAD and UGT72E genes). PAL is the key and start enzyme in the phenylpropanoid pathway and controls the flux of precursors into the phenol network [49]. Both PAL activity and PAL expression are closely related to the accumulation of phenolic acid [50,51]. This study showed that the expression levels of the PAL gene followed an opposite trend to that of phenylalanine but showed the same trend as the content of synthetic product cinnamic acid. These results further confirm that PAL is related to the accumulation of cinnamic acid. C4H is the second key enzyme in the general phenylpropanoid pathway and in addition to PAL, C4H determines the flux through the phenylpropanoid metabolism [52]. COMT mediates the metabolism of coumaric acid to ferulic acid [53] and is also involved in the formation of sinapoyl residues and S-lignin from 5-hydroxyferuloyl derivatives [54]. This study identified significant positive or negative correlations between C4H, COMT enzyme genes and metabolites and a similar trend was found between DEGs and metabolites. These results identified the structural genes that play an important role in the phenolic acid biosynthesis pathway in the leaves of *C. paliurus* at different developmental stages.

TFs are necessary for the regulation of gene expression [55]. Normally, TF proteins function through the combination of their own DNA-binding domain and the cis-acting element of their target genes [56,57]. TFs are critical proteins that regulate gene expression and signal transduction networks during plant growth and development [58]. They also participate in various biological process, including developmental regulation, defense elicitation and stress responses [59,60,61,62]. Based on correlation network analysis, a total of 128 TFs were identified to be related to guiding genes. The three largest families of TFs, APETALA2/ethylene responsive element binding protein (AP2/EREBP), MYB-(R1)R2R3 and bHLH, play a variety of roles throughout the plant life-cycle [63]. This study identified 12 MYB and 10 bHLH TFs. Although it has been considered that only MYB or bHLH could regulate the phenylpropanoid metabolism and enhance the production of secondary metabolites, many studies confirmed that they can play more prominent roles in the form of a complex (e.g., MBW, MYB/bHLH/WD40 protein complex) [64,65,66]. This study showed that the selected MYB and bHLH TFs have similar expression trends and are also similar to the changes of structural genes and metabolite content. This indicates that MYB and bHLH TFs regulate the phenolic acid biosynthesis pathway. Research has shown that the MYB/bHLH complex may significantly elevate the production of secondary metabolites [67]. Based on the phylogenetic tree constructed between the screened MYB and bHLH gene and the *Arabidopsis* R2R3-MYB and bHLH TF (Figures S1 and S2), we suggested that TRINITY_DN94789_c0_g2 MYB, TRINITY_DN94332_c1_g4 MYB, TRINITY_DN97140_c3_g1 bHLH and TRINITY_DN85072_c1_g3 bHLH genes may have the function in regulating the phenolic acid biosynthesis in the leaves of *C. paliurus* during different developmental stages. The possible reason might be due to the clear function of the homologous *Arabidopsis thaliana* MYB TFs (Figure S1, green cluster) and bHLH TFs (Figure S2, blue cluster). Heterologous expression in *Salvia miltiorrhiza* of either snapdragon *ROSEA*1 or *A. thaliana* MYB75/PAP1 boosts the level of both rosmarinic and salvianolic acids. This showed that AtPAP1 was a positive transcriptional activator of the phenolic acid biosynthesis [68,69]. Overexpression of PAP1, *ROSEA*1, *Vv*MYB5a or *StAN*1 resulted in elevated expressions of the genes encoding enzymes for PAL, C4H, 4CL, HCT, COMT and CAD [69]. The reported MYB75, MYB90, MYB111, MYB113 and MYB114 were identified to have regulatory function in the phenylpropanoid biosynthesis pathway [70]. Both of TRINITY_DN94789_c0_g2 and TRINITY_DN94332_c1_g4 MYB genes are highly similar to the CAD, PAL, HCT and COMT gene expression trends and showed significant correlations. bHLH1 showed a strong association with phenylpropanoid expression and the expression patterns of AN1, bHLH1 and WD40 suggest that they are determinants of the amounts of phenylpropanoids that a given genotype will contain [40]. These results indicate that the above two MYB TFs and two bHLH TFs may regulate the expression of these four enzyme genes and thus affect the accumulation of phenolic acids.

Although the other five groups of *Arabidopsis* MYB genes (Figure S1) and all the nine groups of *Arabidopsis* bHLH genes (Figure S2) have not been reported to be involved in the regulation of the phenylpropanoid pathway, they have other functions. For example, AtMYB59 regulates the plant root development by controlling the cell-cycle progression at the root tips [71] and AtMYB99 controls anther development and/or functionality [72]. Both AtMYB88 and AtMYB124/FLP act on stomatal differentiation and patterning by restricting divisions late in the stomatal cell lineage and inducing terminal differentiation [73]. AtbHLH113 in *Arabidopsis* interacts with PAP1/MYB75 modulating anthocyanin biosynthesis [74]. Studies showed that TFs have functional redundancy [75]. However, the function of these TFs should be verified experimentally. Further research is required to identify whether these MYB and bHLH TFs play a regulatory role in the phenolic acid biosynthetic pathway, for instance by chromatin immunoprecipitation assay or by over expressing the candidate TF in plant.

## 4. Materials and Methods

### 4.1. Plant Materials

On May 1, 2018, different developmental stages of *C. paliurus* leaves were collected from Zhuzhang village (E 118°48′28,″ N 28°5′57″), Longquan City, Lishui City, Zhejiang Province, China. The developmental stages of *C. paliurus* leaves are defined as follows: youngest leaf at the F1 stage was the smallest fully expanded leaf, while the matured leaf at the F4 stage referred to full leaf enlargement and full leaf thickness; F2 and F3 referred to intermediate developmental stages between F1 and F4 [76]. For each stage, we collected samples from three plants, which yielded three biological replicates for RNA-seq analysis and metabolome analysis. All samples were immediately frozen in liquid nitrogen after collection and stored at −80 °C until further analysis.

### 4.2. Metabolite Extraction, Multiple Reactions Monitoring and Parameter Setting

Multiple reactions monitoring (MRM) was performed by Metware Biotechnology Co., Ltd. (Wuhan, China). Zirconia beads were added to freeze-dried leaves and were then crushed for 1.5 min at 30 Hz, using a mixing mill (MM400, Retsch, Haan, Germany). 100 mg of the powder was weighed and 1 mL of a 70% aqueous methanol solution was added, followed by extraction overnight at 4 °C. Following centrifugation at 10000 × *g* for 10 min, the extracts were absorbed (CNWBONDCarbon-GCB SPE Cartridge, 250 mg, 3 mL; ANPEL, Shanghai, China, www.anpel.com.cn/cnw) and filtrated (SCAA-104, 0.22 μm pore size; ANPEL, Shanghai, China, http://www.anpel.com.cn/) prior to LC-MS analysis. Then, the sample extracts were analyzed using an LC-ESI-MS/MS system (HPLC, Shim-pack UFLC SHIMADZU CBM30A system, www.shimadzu.com.cn/; MS, Applied Biosystems 6500 Q TRAP, www.appliedbiosystems.com.cn/). The following analytical conditions were used: HPLC: column, Waters ACQUITY UPLC HSS T3 C18 (1.8 µm, 2.1 mm × 100 mm); solvent system, water (0.04% acetic acid): acetonitrile (0.04% acetic acid); gradient program, 95:5 *v/v* at 0 min, 5:95 *v/v* at 11 min, 5:95 *v/v* at 12 min, 95:5 *v/v* at 12.1 min, 95:5 *v/v* at 15 min; flow rate, 0.4 mL/min; temperature, 40 °C; injection volume: 2 μL. The effluent was alternatively connected to an ESI-triple quadrupole-linear ion trap (Q TRAP)-MS. Linear ion trap (LIT) and triple quadrupole (QQQ) scans were acquired on a triple quadrupole-linear ion trap mass spectrometer (Q TRAP), API 6500 Q TRAP LC/MS/MS System, equipped with an ESI Turbo Ion-Spray interface, operated in positive ion mode and controlled by Analyst 1.6 software (AB Sciex, Concord, Ontario, Canada). The following ESI source operation parameters were used: ion source: turbo spray; source temperature: 500 °C; ion spray voltage (IS): 5500 V; ion source gas I (GSI), gas II (GSII) and curtain gas (CUR) were set to 55, 60 and 25.0 psi, respectively; the collision gas (CAD) was high. Instrument tuning and mass calibration were performed with 10 and 100 μmol/L polypropylene glycol solutions in QQQ and LIT modes, respectively. QQQ scans were acquired as MRM experiments with collision gas (nitrogen) at 5 psi. The declustering potential (DP) and collision energy (CE) for individual MRM transitions were measured with further DP and CE optimization. A specific set of MRM transitions was monitored for each period according to the metabolites that were eluted within this period. Metabolites with significant differences in content were defined as having a variable importance in project (VIP) ≥1 and a fold change of ≥ 2 or ≤ 0.5. The MRM for each cultivar was performed in triplicate. Three spears were used for each repetition.

### 4.3. Transciptomic Analysis

The total RNA was extracted from each sample using the Total RNA Extractor (Trizol) kit (B511311, Sangon, Shanghai, China) according to the manufacturer’s protocol. After the removal of the genomic DNA contaminations by the digestion of DNase I. Three cDNA libraries were generated for every developmental stage using the VAHTSTM mRNA-seq V2 Library Prep Kit (Illumina, San Diego, CA, USA) following the manufacturer’s protocols. In total, 12 cDNA library were constructed during four developmental stages. Index codes were added to attribute sequences to the specific samples. The first strand cDNA was synthesized using random hexamer primer and M-MuLV Reverse Transcriptase (RNase H-), while the second strand cDNA synthesis was subsequently performed using DNA polymerase I and RNase H. Remaining overhangs were converted into blunt ends via exonuclease/polymerase activities. Library fragments were purified with the AMPure XP system (Beckman Coulter Company, Beverly, MA, USA). Polymerase chain reactions (PCR) were performed with Phusion High-Fidelity DNA polymerase (Thermo Fisher Scientific Inc., Waltham, MA, USA), universal PCR primers and Index (X) Primer. The library quality was assessed on the Bioanalyzer 2100 system (Agilent Technologies Inc. Santa Clara, CA, USA). After quantifying and pooling, paired-end sequencing of these libraries was performed on HiSeq XTen sequencers (Illumina, San Diego, CA, USA) by Novogen Co., Ltd. (Beijing, China).

Raw data or raw reads of high-throughput sequencing, including gene ID and sequence, were stored in the FASTA file format. Raw reads were filtered by Trimmomatic (version 0.36) to gain clean reads, which were *de novo* assembled into transcripts using Trinity (version 2.0.6) (parameter: min_kmer_cov 2) (Trinity Technologies, Irvine, CA, USA). Transcripts with a minimum length of 200 bp were clustered to decrease redundancy. For each cluster, the one with the longest region of high-quality sequence data was selected as a Unigene. Unigenes were blasted and annotated against NCBI Nr (NCBI non-redundant protein database), SwissProt, TrEMBL, CDD (Conserved Domain Database), Pfam and KOG (eukaryotic Orthologous Groups) databases (E-value < 1e-5). According to the priority of the best alignment results, the corresponding amino acid sequence of Unigene ORF was determined. At the same time, TransDecoder (version 3.0.1) was used to predict the CDS sequences of the unaligned Unigene. According to the annotation results of SwissProt and TrEMBL, GO (Gene Ontology Database) function annotation information was obtained. KEGG (Kyoto Encyclopedia of Genes and Genomes) annotation was conducted by KAAS (version 2.1) (KEGG automatic annotation server).

### 4.4. Differentially Expressed Genes Analysis

The quality control sequence was mapped to the assembled transcript using Bowtie 2 (version 2.3.2). The statistical analysis of the alignment results used RSeQC (version 2.6.1). The count and expression values of single gene readings were calculated using salmon (version 0.8.2). Transcripts per million (TPM) was used to eliminate the influence of gene lengths and sequencing discrepancies, enabling direct comparison of gene expressions between samples. The differentially expressed genes (DEGs) between the two samples were identified using DESeq2 (version 1.12.4). Genes were considered at a q-value < 0.001 and |FoldChange| > 2. When the normalized expression of a gene was zero between two samples, its expression value was adjusted to 0.01 (since 0 cannot be displayed on a log plot). If the normalized expression of a specific gene in two libraries was lower than 1, the further differential expression analysis was conducted excluding this gene.

### 4.5. Real-Time Quantitative PCR

Six candidate genes, six MYB and six bHLH transcription factor genes, involved in phenolic acid biosynthesis, were selected for real-time quantitative PCR verification. Real-time PCR was performed on CFX Connect (Bio-Rad Laboratories Inc. Hercules, CA, USA) by using HiScript II reverse transcriptase according to the manufacturer’s protocols (Vazyme Biotech Co. Ltd., Nanjing, China). The specific primers for glycosyltransferase genes were designed using Primer Premier 5.0 (Supplementary material 5). A constitutively expressed gene (*β-Actin-1*) was used as internal control [77]. The relative expression values of genes were calculated as 2^−∆∆Ct^. Three biological replicates and three technical replicates were used for all qRT-PCRs.

### 4.6. Transcription Factors Prediction and Analysis

Transcription factors were predicted with PlantTFDB [78]. The correlation coefficient between genes and metabolites and between genes and transcription factors, was calculated with the corrplot package in R-3.6.1 and the correlation network was visualized using Cytoscape software (version 3.6.1). The phylogenetic tree of the predicted transcription factors and the known transcription factors in *Arabidopsis thialiana* was generated with 2000 bootstrap replicates by the neighbor-joining method using software Mega X ver. 10.0.2.

### 4.7. Statistical Analysis

The data are displayed as means ± standard deviations (SD). One-way analysis of variance (ANOVA) was used to evaluate the significance at the 0.05 level. All statistical analyses were conducted by SPSS 17.0 software (SPSS Inc., Chicago, IL USA).

### 4.8. Accession Numbers

All raw data have been deposited at the National Center for Biotechnology Information (NCBI) database (under the BioProject accession number PRJNA 548403).

## 5. Conclusions

This study used a combination of metabolome and transcriptome to interpret the relationships between key genes and metabolites involved in biosynthetic pathways. Both candidate genes and metabolites were identified that are involved in the phenolic acid biosynthetic pathway. The candidate genes include six structural genes (*PAL*, *C4H*, *HCT*, *CAD*, *COMT* and *UGT72E*), 12 MYB TFs and 10 bHLH TFs. Although these genes were predicted by the bioinformatics analysis, the validation of the transcriptomic expression were verified by qRT-PCR and these candidate genes provide valuable information and useful references to better understand the regulation of the phenolic acid biosynthesis pathway and the accumulation of metabolites in the leaves of *C. paliurus* during different developmental stages. Nevertheless, the specific mechanism still requires further research.

## Figures and Tables

**Table 1:** Content and type of metabolites in phenolic acid biosynthesis pathway.

Compounds	Molecular Weight (Da)	CPS			
		F1	F2	F3	F4
Sinapoyl malate	340.1	180000 ± 80600b	85400 ± 36800b	74600 ± 14700b	441000 ± 97800a
Caffeic aldehyde	164.1	175000 ± 11600a	238000 ± 72700a	237000 ± 26300a	196000 ± 44500a
L-Phenylalanine	165.079	25700000 ± 1560000b	26600000 ± 5000000b	34200000 ± 3860000a	3200000 ± 647000c
Cinnamic acid	148.0524	71000 ± 13900a	98900 ± 16200a	79700 ± 16800a	126000 ± 77500a
Caffeic acid	180.0423	378000 ± 40700b	772000 ± 384000ab	608000 ± 47600ab	1700000 ± 891000a
Ferulic acid	194.0579	8430000 ± 6070000a	7460000 ± 3860000a	9710000 ± 4460000a	9460000 ± 3640000a
Chlorogenic acid	354.0951	3060000 ± 237000a	2600000 ± 318000a	2520000 ± 267000a	1800000 ± 146000b
Coniferylaldehyde	178.063	2090000 ± 622000b	1930000 ± 470000b	3050000 ± 106000a	1880000 ± 264000b
Syringin	372.142	13300 ± 15000b	60300 ± 21800b	33500 ± 23700b	118000 ± 32500a
p-Coumaric acid	164.047	368000 ± 131000a	295000 ± 180000a	84700 ± 34900a	591000 ± 598000a
Coniferyl alcohol	180.079	96900 ± 48600b	110000 ± 39100b	65700 ± 15700b	502000 ± 54300a
Sinapic acid	224.069	15500 ± 7880b	41200 ± 25300b	19000 ± 12900b	457000 ± 183000a
Sinapyl alcohol	210.089	6040 ± 412b	8300 ± 6100b	1180 ± 1660b	103000 ± 8870a
Coniferin	342.132	1660000 ± 2350000a	21900000 ± 17300000a	5200000 ± 7350000a	44700000 ± 40500000a

* CPS, Count Per Second; SE, Standard Error. Different small letters indicate significant difference among four different developmental stages.

## Supplementary Materials

Supplementary materials can be found at https://www.mdpi.com/1422-0067/21/4/1337/s1.

## References

[1] Li A.N., Li S., Zhang Y.J., Xu X.R., Chen Y.M., Li H.B. (2014). Resources and biological activities of natural polyphenols. Nutrients.

[2] Ma D.Y., Li Y.G., Zhang J., Wang C.Y., Qin H.X., Ding H.N., Xie Y.X., Guo T.C. (2016). Accumulation of phenolic compounds and expression profiles of phenolic acid biosynthesis-related genes in developing grains of white, purple and red wheat. Front. Plant Sci..

[3] Ozdal T., Sela D.A., Xiao J., Boyacioglu D., Chen F., Capanoglu E. (2016). The reciprocal interactions between polyphenols and gut microbiota and effects on bioaccessibility. Nutrients.

[4] Ambrizpérez D.L., Leyvalópez N., Gutierrezgrijalva E.P., Heredia J.B. (2016). Phenolic compounds: Natural alternative in inflammation treatment. A review. Cogent Food Agric..

[5] Azam S., Hadi N., Khan N.U., Hadi S.M. (2004). Prooxidant property of green tea polyphenols epicatechin and epigallocatechin-3-gallate: Implications for anticancer properties. Toxicol. In Vitro.

[6] Mattila P., Pihlava J.M., Hellstrom J. (2005). Contents of phenolic acids, alkyl- and alkenylresorcinols and avenanthramides in commercial grain products. J. Agric. Food Chem..

[7] Mandal S.M., Chakraborty D., Dey S. (2010). Phenolic acids act as signaling molecules in plant-microbe symbioses. Plant Signal Behav..

[8] Meskin M.S., Bidlack W.R., Davies A.J., Omaye S.T. (2002). Phytochemicals in Nutrition and Health.

[9] Herrmann K. (1989). Occurrence and content of hydroxycinnamic and hydroxybenzoic acid compounds in foods. Crit. Rev. Food Sci..

[10] Gross G.G. (1985). CHAPTER 10—Biosynthesis and metabolism of phenolic acids and monolignols. Biosynthesis and Biodegradation of Wood Components.

[11] Heleno S.A., Martins A., Queiroz M.J.R.P., Ferreira I.C.F.R. (2015). Bioactivity of phenolic acids: Metabolites versus parent compounds: A review. Food Chem..

[12] Kumar N., Goel N. (2019). Phenolic acids: Natural versatile molecules with promising therapeutic applications. Biotechnol. Rep..

[13] Deng Y.X., Lu S.F. (2017). Biosynthesis and regulation of phenylpropanoids in plants. Crit. Rev. Plant Sci..

[14] Nile S.H., Park S.W. (2014). Edible berries: Bioactive components and their effect on human health. Nutrition.

[15] Neacsu M., Vaughan N., Raikos V., Multari S., Duncan G.J., Duthie G.G., Russell W.R. (2015). Phytochemical profile of commercially available food plant powders: Their potential role in healthier food reformulations. Food Chem..

[16] Mehrabani L.V., Hassanpouraghdam M.B. (2012). Developmental variation of phenolic compounds in fruit tissue of two apple cultivars. Acta Sci. Polonorum Technol. Aliment..

[17] Ding C.K., Chachin K., Ueda Y., Imahori Y., Wang C.Y. (2001). Metabolism of phenolic compounds during loquat fruit development. J. Agric. Food Chem..

[18] Payyavula R.S., Navarre D.A., Joseph K., Alberto P. (2013). Developmental effects on phenolic, flavonol, anthocyanin and carotenoid metabolites and gene expression in potatoes. J. Agric. Food Chem..

[19] Fiehn O., Kopka J., Dormann P., Altmann T., Trethewey R.N., Willmitzer L. (2000). Metabolite profiling for plant functional genomics. Nat. Biotechnol..

[20] Sumner L.W., Mendes P., Dixon R.A. (2003). Plant metabolomics: Large-scale phytochemistry in the functional genomics era. Phytochemistry.

[21] Fukushima A., Kusano M., Redestig H. (2009). Integrated omics approaches in plant systems biology. Curr. Opin. Chem. Biol..

[22] van Assche R., Broeckx V., Boonen K., Maes E., de Haes W., Schoofs L., Temmerman L. (2015). Integrating-omics: Systems biology as explored through C-elegans research. J. Mol. Biol..

[23] Li Y.K., Fang J.B., Qi X.J., Lin M.M., Zhong Y.P., Sun L.M., Cui W. (2018). Combined analysis of the fruit metabolome and transcriptome reveals candidate genes involved in flavonoid biosynthesis in *Actinidia arguta*. Int. J. Mol. Sci..

[24] Kleessen S., Irgang S., Klie S., Giavalisco P., Nikoloski Z. (2015). Integration of transcriptomics and metabolomics data specifies Chlamydomonas’ metabolic response to rapamycin tratment. Plant J..

[25] Glaubitz U., Li X., Schaedel S., Erban A., Sulpice R., Kopka J., Hincha D.K., Zuther E. (2017). Integrated analysis of rice transcriptomic and metabolomic responses to elevated night temperatures identifies sensitivity- and tolerance-related profiles: Integrated profiling analysis of rice under HNT. Plant Cell Environ..

[26] Lou Q., Liu Y., Qi Y., Jiao S., Tian F., Jiang L., Wang Y. (2014). Transcriptome sequencing and metabolite analysis reveals the role of delphinidin metabolism in flower colour in grape hyacinth. J. Exp. Bot..

[27] Cho K., Cho K.S., Sohn H.B., Ha I.J., Hong S.Y., Lee H., Kim Y.M., Nam M.H. (2016). Network analysis of the metabolome and transcriptome reveals novel regulation of potato pigmentation. J. Exp. Bot..

[28] Wu Q., Wu J., Li S.S., Zhang H.J., Feng C.Y., Yin D.D., Wu R.Y., Wang L.S. (2016). Transcriptome sequencing and metabolite analysis for revealing the blue flower formation in waterlily. BMC Genom..

[29] Liu G.F., Han Z.X., Feng L., Gao L.P., Gao M.J., Gruber M.Y., Zhang Z.L., Xia T., Wan X.C., Wei S. (2017). Metabolic flux redirection and transcriptomic reprogramming in the albino tea cultivar ‘Yu-Jin-Xiang’ with an emphasis on catechin production. Sci. Rep..

[30] Zhu G.T., Wang S.C., Huang Z.J., Zhang S.B., Liao Q.G., Zhang C.Z., Lin T., Qin M., Peng M., Yang C.K. (2018). Rewiring of the fruit metabolome in tomato breeding. Cell.

[31] Hirai M.Y., Yano M., Goodenowe D.B., Kanaya S., Kimura T., Awazuhara M., Arita M., Fujiwara T., Saito K. (2004). Integration of transcriptomics and metabolomics for understanding of global responses to nutritional stresses in *Arabidopsis thaliana*. Proc. Natl. Acad. Sci. USA.

[32] Liu Y., Qian C.Y., Ding S.H., Shang X.L., Yang W.X., Fang S.Z. (2016). Effect of light regime and provenance on leaf characteristics, growth and flavonoid accumulation in *Cyclocarya paliurus* (Batal) Iljinskaja coppices. Bot. Stud..

[33] Xie J.H., Liu X., Shen M.Y., Nie S.P., Zhang H., Li C., Gong D.M., Xie M.Y. (2013). Purification, physicochemical characterisation and anticancer activity of a polysaccharide from *Cyclocarya paliurus* leaves. Food Chem..

[34] Yang Z.W., Wang J., Li J.G., Xiong L., Chen H., Liu X., Wang N., Ouyang K.H., Wang W.J. (2018). Antihyperlipidemic and hepatoprotective activities of polysaccharide fraction from *Cyclocarya paliurus* in high-fat emulsion-induced hyperlipidaemic mice. Carbohydr. Polym..

[35] Xie J.H., Dong C.J., Nie S.P., Li F., Wang Z.J., Shen M.Y., Xie M.Y. (2015). Extraction, chemical composition and antioxidant activity of flavonoids from *Cyclocarya paliurus* (Batal.) Iljinskaja leaves. Food Chem..

[36] Kurihara H., Asami S., Shibata H., Fukami H., Tanaka T. (2003). Hypolipemic effect of *Cyclocarya paliurus* (Batal) Iljinskaja in lipid-loaded mice. Biol. Pharm. Bull..

[37] Wang Z.J., Xie J.H., Kan L.J., Wang J.Q., Shen M.Y., Li W.J., Nie S.P., Xie M.Y. (2015). Sulfated polysaccharides from *Cyclocarya paliurus* reduce H_2_O_2_-induced oxidative stress in RAW264.7 cells. Int. J. Biol. Macromol..

[38] Cao Y.N., Fang S.Z., Fu X.X., Shang X.L., Yang W.X. (2019). Seasonal variation in phenolic compounds and antioxidant activity in leaves of *Cyclocarya paliurus* (Batal.) Iljinskaja. Forests.

[39] Bo D., Fang S.Z., Shang X.L., Fu X.X., Yan L. (2019). Influence of provenance and shade on biomass production and triterpenoid accumulation in *Cyclocarya paliurus*. Agrofor. Syst..

[40] Payyavula R.S., Singh R.K., Navarre D.A. (2013). Transcription factors, sucrose and sucrose metabolic genes interact to regulate potato phenylpropanoid metabolism. J. Exp. Bot..

[41] Zhao S.C., Tuan P.A., Li X.H., Kim Y.B., Kim H.R., Park C.G., Yang J.L., Li C.H., Park S.U. (2013). Identification of phenylpropanoid biosynthetic genes and phenylpropanoid accumulation by transcriptome analysis of *Lycium chinense*. BMC Genom..

[42] Gayoso C., Pomar F., Novo Uzal E., Merino F., de Ilarduya O.M. (2010). The *Ve*-mediated resistance response of the tomato to *Verticillium dahliae* involves H_2_O_2_, peroxidase and lignins and drives *PAL* gene expression. BMC Plant Biol..

[43] Jiang X.L., Liu Y.J., Li W.W., Zhao L., Meng F., Wang Y.S., Tan H.R., Yang H., Wei C.L., Wan X.C. (2013). Tissue-specific, development-dependent phenolic compounds accumulation profile and gene expression pattern in tea plant (*Camellia sinensis*). PLoS ONE.

[44] Qu C.M., Fu F.Y., Lu K., Zhang K., Wang R., Xu X.F., Wang M., Lu J.X., Wan H.F., Tang Z.L. (2013). Differential accumulation of phenolic compounds and expression of related genes in black- and yellow-seeded *Brassica napus*. J. Exp. Bot..

[45] Guo H.H., Guo H.X., Zhang L., Tang Z.M., Yu X.M., Wu J.F., Zeng F.C. (2019). Metabolome and transcriptome association analysis reveals dynamic regulation of purine metabolism and flavonoid synthesis in transdifferentiation during somatic embryogenesis in cotton. Int. J. Mol. Sci..

[46] Hall R., Beale M., Fiehn O., Hardy N., Sumner L., Bino R. (2002). Plant metabolomics: The missing link in functional genomics strategies. Plant Cell.

[47] Fiehn O. (2002). Metabolomics—The link between genotypes and phenotypes. Plant Mol. Biol..

[48] Singh A., Desgagne Penix I. (2017). Transcriptome and metabolome profiling of *Narcissus pseudonarcissus* ‘King Alfred’ reveal components of Amaryllidaceae alkaloid metabolism. Sci. Rep..

[49] Liu C.H., Zheng H.H., Sheng K.L., Liu W., Zheng L. (2018). Effects of postharvest UV-C irradiation on phenolic acids, flavonoids and key phenylpropanoid pathway genes in tomato fruit. Sci. Hortic..

[50] Mccallum J.A., Walker J.R.L. (1990). Phenolic biosynthesis during grain development in wheat: Changes in phenylalanine ammonia-lyase activity and soluble phenolic content. J. Cereal Sci..

[51] Andre C.M.S.R., Legay S., Lefevre I., Alvarado Aliaga C.A., Nomberto G., Hoffmann L., Hausman J.F., Larondelle Y., Evers D. (2009). Gene expression changes related to the production of phenolic compounds in potato tubers grown under drought stress. Phytochemistry.

[52] Blount J.W., Korth K.L., Masoud S.A., Rasmussen S., Lamb C., Dixon R.A. (2000). Altering expression of cinnamic acid 4-hydroxylase in transgenic plants provides evidence for a feedback loop at the entry point into the phenylpropanoid pathway. Plant Physiol..

[53] Boerjan W., Ralph J., Baucher M. (2003). Lignin biosynthesis. Annu. Rev. Plant Biol..

[54] Davin L.B., Jourdes M., Patten A.M., Kim K.W., Vassão D.G., Lewis N.G. (2008). Dissection of lignin macromolecular configuration and assembly: Comparison to related biochemical processes in allyl/propenyl phenol and lignan biosynthesis. Cheminform.

[55] Salih H., Gong W.F., He S.P., Sun G.F., Sun J.L., Du X.M. (2016). Genome-wide characterization and expression analysis of MYB transcription factors in *Gossypium hirsutum*. BMC Genet..

[56] Valentina B. (2016). Analysis of genomic sequence motifs for deciphering transcription factor binding and transcriptional regulation in eukaryotic cells. Front. Genet..

[57] Orenstein Y., Shamir R. (2017). Modeling protein–DNA binding via high-throughput in vitro technologies. Brief. Funct. Genom..

[58] Miao L., Di Q.H., Sun T.S., Li Y.S., Duan Y., Wang J., Yan Y., He C.X., Wang C.L., Yu X.C. (2019). Integrated metabolome and transcriptome analysis provide insights into the effects of grafting on fruit flavor of cucumber with different rootstocks. Int. J. Mol. Sci..

[59] Shiu S.H., Shih M.C., Li W.H. (2005). Transcription factor families have much higher expansion rates in plants than in animals. Plant Physiol..

[60] Schwechheimer C., Bevan M. (1998). The regulation of transcription factor activity in plants. Trends Plant Sci..

[61] Wong D.C.J., Schlechter R., Vannozzi A., Hoell J., Hmmam I., Bogs J., Tornielli G.B., Castellarin S.D., Tomas Matus J. (2016). A systems-oriented analysis of the grapevine R2R3-MYB transcription factor family uncovers new insights into the regulation of stilbene accumulation. DNA Res..

[62] Zhang X.R., Dong J.N., Liu H.L., Wang J., Qi Y.X., Liang Z.S. (2016). Transcriptome sequencing in response to salicylic acid in *Salvia miltiorrhiza*. PLoS ONE.

[63] Riechmann J.L., Ratcliffe O.J. (2000). A genomic perspective on plant transcription factors. Curr. Opin. Plant Biol..

[64] Gonzalez A., Zhao M., Leavitt J.M., Lloyd A.M. (2008). Regulation of the anthocyanin biosynthetic pathway by the TTG1/bHLH/Myb transcriptional complex in *Arabidopsis* seedlings. Plant J..

[65] Gonzalez A. (2009). Pigment loss in response to the environment: A new role for the WD/bHLH/MYB anthocyanin regulatory complex. New Phytol..

[66] Qi T.C., Song S.S., Ren Q.C., Wu D.W., Huang H., Chen Y., Fan M., Peng W., Ren C.M., Xie D.X. (2011). The Jasmonate-ZIM-domain proteins interact with the WD-Repeat/bHLH/MYB complexes to regulate Jasmonate-mediated anthocyanin accumulation and trichome initiation in *Arabidopsis thaliana*. Plant Cell.

[67] Wang D.H., Song Y., Chen Y.Q., Yao W., Li Z., Liu W.C., Yue S.S., Wang Z.Z. (2013). Metabolic pools of phenolic acids in *Salvia miltiorrhiza* are enhanced by co-expression of *Antirrhinum majus Delila* and *Rosea1* transcription factors. Biochem. Eng. J..

[68] Zhang Y., Yan Y.P., Wang Z.Z. (2010). The Arabidopsis PAP1 transcription factor plays an important role in the enrichment of phenolic acids in *Salvia miltiorrhiza*. J. Agric. Food Chem..

[69] Liu J., Osbourn A., Ma P. (2015). MYB Transcription factors as regulators of phenylpropanoid metabolism in plants. Mol. Plant.

[70] Mondal S.K., Roy S. (2018). Genome-wide sequential, evolutionary, organizational and expression analyses of phenylpropanoid biosynthesis associated MYB domain transcription factors in *Arabidopsis*. J. Biomol. Struct. Dyn..

[71] Mu R.L., Cao Y.R., Liu Y.F., Lei G., Zou H.F., Liao Y., Wang H.W., Zhang W.K., Ma B., Du J.Z. (2009). An R2R3-type transcription factor gene *AtMYB59* regulates root growth and cell cycle progression in *Arabidopsis*. Cell Res..

[72] Battat M., Eitan A., Rogachev I., Hanhineva K., Fernie A., Tohge T., Beekwilder J., Aharoni A. (2019). A MYB triad controls primary and phenylpropanoid metabolites for pollen coat patterning. Plant Physiol..

[73] Lai L.B., Nadeau J.A., Jessica L., Eun-Kyoung L., Tsuyoshi N., Liming Z., Matt G., Sack F.D. (2005). The *Arabidopsis* R2R3 MYB proteins FOUR LIPS and MYB88 restrict divisions late in the stomatal cell lineage. Plant Cell.

[74] Shin D.H., Cho M., Choi M.G., Das P.K., Lee S.K., Choi S.B., Park Y.I. (2015). Identification of genes that may regulate the expression of the transcription factor production of anthocyanin pigment 1 (PAP1)/MYB75 involved in Arabidopsis anthocyanin biosynthesis. Plant Cell Rep..

[75] Wang A.M., Li R.S., Ren L., Gao X.L., Zhang Y.G., Ma Z.M., Ma D.F., Luo Y.H. (2018). A comparative metabolomics study of flavonoids in sweet potato with different flesh colors (*Ipomoea batatas* (L.) Lam). Food Chem..

[76] Guo F., Guo Y.F., Wang P., Wang Y., Ni D.J. (2017). Transcriptional profiling of catechins biosynthesis genes during tea plant leaf development. Planta.

[77] Jin J.P., Tian F., Yang D.C., Meng Y.Q., Kong L., Luo J.C., Gao G. (2017). PlantTFDB 4.0: Toward a central hub for transcription factors and regulatory interactions in plants. Nucleic Acids Res..

[78] Zhao S., Zhang X.M., Su Y.P., Chen Y.L., Liu Y., Sun M., Qi G.H. (2018). Transcriptome analysis reveals dynamic fat accumulation in the walnut kernel. Int. J. Genom..

