# Supplementary material for: Combined Analysis of the Metabolome and Transcriptome Identified Candidate Genes Involved in Phenolic Acid Biosynthesis in the Leaves of Cyclocarya paliurus
Source: Int J Mol Sci. 2020 Feb 17;21(4):1337. doi: 10.3390/ijms21041337 (PMC7073005; doi:10.3390/ijms21041337)
Supplement: Supplementary file 1 [file ijms-21-01337-s001.pdf]

Supplementary material 1 Correlation between genes and metabolites (P-value≤0.05, PCC≥0.9)

Gene ID	metabolites	P-value	PCC
TRINITY_DN150009_c0_g1(PAL)	L-Phenylalanine	0.006674058	-0.993325942
TRINITY_DN150009_c0_g1(PAL)	Coniferyl alcohol	0.030738028	0.969261972
TRINITY_DN150009_c0_g1(PAL)	Sinapic acid	0.037617847	0.962382153
TRINITY_DN205624_c0_g1(PAL)	L-Phenylalanine	0.031888457	0.968111543
TRINITY_DN205624_c0_g1(PAL)	Chlorogenic acid	0.026049843	0.973950157
TRINITY_DN205624_c0_g1(PAL)	Sinapic acid	0.025225938	-0.974774062
TRINITY_DN63813_c0_g1(4CL)	Coniferylaldehyde	0.009072645	-0.990927355
TRINITY_DN63813_c0_g1(4CL)	p-Coumaric acid	0.018375407	0.981624593
TRINITY_DN76182_c0_g2(C4H)	Coniferin	0.037333347	-0.962666653
TRINITY_DN80908_c0_g1(CAD)	Caffeic acid	0.032503295	-0.967496705
TRINITY_DN80908_c0_g1(CAD)	Chlorogenic acid	4.34E-04	0.999566049
TRINITY_DN82370_c0_g1(CAD)	L-Phenylalanine	0.006403104	0.993596896
TRINITY_DN82370_c0_g1(CAD)	Coniferyl alcohol	0.027871029	-0.972128971
TRINITY_DN82370_c0_g1(CAD)	Sinapic acid	0.031253042	-0.968746958
TRINITY_DN82952_c2_g2(HCT)	L-Phenylalanine	0.023026237	0.976973763
TRINITY_DN83539_c2_g6(C4H)	Coniferylaldehyde	0.036224255	-0.963775745
TRINITY_DN83539_c2_g6(C4H)	p-Coumaric acid	0.00167527	0.99832473
TRINITY_DN84482_c0_g8(4CL)	Sinapoyl malate	0.024114584	0.975885416
TRINITY_DN84482_c0_g8(4CL)	L-Phenylalanine	0.025735537	-0.974264463
TRINITY_DN84482_c0_g8(4CL)	Coniferyl alcohol	0.047995552	0.952004448
TRINITY_DN85465_c1_g1(HCT)	Sinapoyl malate	0.011265251	0.988734749
TRINITY_DN85751_c2_g2(E2.1.1.104)	p-Coumaric acid	0.021188106	0.978811894
TRINITY_DN86495_c2_g1(4CL)	Sinapoyl malate	0.028885232	0.971114768
TRINITY_DN86835_c0_g2(F5H)	Caffeic acid	0.029800821	0.970199179
TRINITY_DN86835_c0_g2(F5H)	Chlorogenic acid	0.0024897	-0.9975103
TRINITY_DN86835_c0_g2(F5H)	Sinapic acid	0.037453055	0.962546945
TRINITY_DN87383_c2_g1(PAL)	Sinapoyl malate	0.037975494	0.962024506
TRINITY_DN87383_c2_g1(PAL)	Coniferyl alcohol	0.037193559	0.962806441
TRINITY_DN87383_c2_g1(PAL)	Sinapyl alcohol	0.020665901	0.979334099
TRINITY_DN88568_c2_g1(E2.1.1.104)	p-Coumaric acid	0.015339428	0.984660572
TRINITY_DN88568_c2_g3(E2.1.1.104)	L-Phenylalanine	0.013664837	-0.986335163
TRINITY_DN88568_c2_g3(E2.1.1.104)	Coniferyl alcohol	0.003829138	0.996170862
TRINITY_DN88568_c2_g3(E2.1.1.104)	Sinapyl alcohol	0.023278992	0.976721008
TRINITY_DN89592_c0_g1(4CL)	Ferulic acid	0.028380224	-0.971619776
TRINITY_DN89841_c2_g8(E2.1.1.104)	Coniferylaldehyde	0.01623983	-0.98376017
TRINITY_DN89841_c2_g8(E2.1.1.104)	p-Coumaric acid	0.030004901	0.969995099
TRINITY_DN92541_c0_g1(CAD)	Coniferyl alcohol	0.022441114	0.977558886
TRINITY_DN92541_c0_g1(CAD)	Sinapic acid	0.021523398	0.978476602
TRINITY_DN92541_c0_g1(CAD)	Sinapyl alcohol	0.032337393	0.967662607

TRINITY_DN92995_c1_g3(4CL)	Coniferylaldehyde	0.017930854	0.982069146
TRINITY_DN93270_c1_g1(UGT72E)	Sinapoyl malate	0.034423545	-0.965576455
TRINITY_DN94191_c0_g2(PAL)	Sinapoyl malate	0.040184278	0.959815722
TRINITY_DN94191_c0_g2(PAL)	L-Phenylalanine	0.041689272	-0.958310728
TRINITY_DN94191_c0_g2(PAL)	Coniferyl alcohol	0.024173794	0.975826206
TRINITY_DN94191_c0_g2(PAL)	Sinapyl alcohol	0.019019957	0.980980043
TRINITY_DN94565_c3_g1(HCT)	Caffeic aldehyde	0.025083993	-0.974916007
TRINITY_DN95472_c1_g2(COMT)	Coniferylaldehyde	0.044979936	-0.955020064
TRINITY_DN95472_c1_g2(COMT)	p-Coumaric acid	0.004638094	0.995361906
TRINITY_DN95628_c5_g2(E2.1.1.104)	p-Coumaric acid	0.036412727	0.963587273
TRINITY_DN95767_c2_g7(C4H)	Caffeic aldehyde	0.026186207	0.973813793
TRINITY_DN95893_c4_g2(PAL)	Coniferylaldehyde	7.37E-04	-0.999262891
TRINITY_DN96670_c1_g3(CCR)	Cinnamic acid	0.016568114	0.983431886
TRINITY_DN96670_c1_g3(CCR)	Caffeic acid	7.75E-04	0.999225086
TRINITY_DN96670_c1_g3(CCR)	Chlorogenic acid	0.026905669	-0.973094331
TRINITY_DN96670_c1_g3(CCR)	Syringin	0.036193113	0.963806887
TRINITY_DN96670_c1_g3(CCR)	Sinapic acid	0.030357641	0.969642359
TRINITY_DN96776_c2_g1(CAD)	Coniferyl alcohol	0.025661412	0.974338588
TRINITY_DN96776_c2_g1(CAD)	Sinapyl alcohol	0.004666435	0.995333565
TRINITY_DN96993_c1_g1(CAD)	p-Coumaric acid	0.026120271	0.973879729
TRINITY_DN96993_c1_g1(CAD)	Sinapyl alcohol	0.028941943	0.971058057
TRINITY_DN97112_c0_g3(HCT)	Coniferylaldehyde	0.030579617	-0.969420383
TRINITY_DN97112_c0_g3(HCT)	p-Coumaric acid	0.00338241	0.99661759
TRINITY_DN97112_c1_g2(HCT)	Coniferylaldehyde	0.045729773	-0.954270227
TRINITY_DN97112_c1_g2(HCT)	p-Coumaric acid	0.00376434	0.99623566

Supplementary material 2 Correlation between guide genes and differentially expressed TFs (P-value ≤ 0.05, PCC ≥ 0.9)

Guide genes	Differentially expressed TFs	P-value	PCC
TRINITY_DN92541_c0_g1(CAD)	TRINITY_DN82329_c0_g1(ERF)	0.023350177	0.976649823
TRINITY_DN87383_c2_g1(PAL)	TRINITY_DN82329_c0_g1(ERF)	0.010266282	0.989733718
TRINITY_DN97112_c1_g2(HCT)	TRINITY_DN82329_c0_g1(ERF)	0.014681988	0.985318012
TRINITY_DN95472_c1_g2(COMT)	TRINITY_DN82329_c0_g1(ERF)	0.019393053	0.980606947
TRINITY_DN92541_c0_g1(CAD)	TRINITY_DN82983_c2_g1(NAC)	0.039747256	0.960252744
TRINITY_DN87383_c2_g1(PAL)	TRINITY_DN82983_c2_g1(NAC)	0.029401544	0.970598456
TRINITY_DN97112_c1_g2(HCT)	TRINITY_DN82983_c2_g1(NAC)	0.037377838	0.962622162
TRINITY_DN95472_c1_g2(COMT)	TRINITY_DN82983_c2_g1(NAC)	0.044646149	0.955353851
TRINITY_DN92541_c0_g1(CAD)	TRINITY_DN83405_c2_g6(ERF)	0.01522259	0.98477741
TRINITY_DN87383_c2_g1(PAL)	TRINITY_DN83405_c2_g6(ERF)	0.017054979	0.982945021
TRINITY_DN97112_c1_g2(HCT)	TRINITY_DN83405_c2_g6(ERF)	0.019054968	0.980945032
TRINITY_DN95472_c1_g2(COMT)	TRINITY_DN83405_c2_g6(ERF)	0.023825621	0.976174379
TRINITY_DN92541_c0_g1(CAD)	TRINITY_DN83568_c0_g1(bHLH)	0.028851566	0.971148434
TRINITY_DN87383_c2_g1(PAL)	TRINITY_DN83568_c0_g1(bHLH)	0.01338091	0.98661909
TRINITY_DN97112_c1_g2(HCT)	TRINITY_DN83568_c0_g1(bHLH)	0.01925256	0.98074744
TRINITY_DN95472_c1_g2(COMT)	TRINITY_DN83568_c0_g1(bHLH)	0.024636079	0.975363921
TRINITY_DN92541_c0_g1(CAD)	TRINITY_DN83568_c0_g3(bHLH)	0.028115092	0.971884908
TRINITY_DN87383_c2_g1(PAL)	TRINITY_DN83568_c0_g3(bHLH)	0.00343808	0.99656192
TRINITY_DN97112_c1_g2(HCT)	TRINITY_DN83568_c0_g3(bHLH)	0.008019355	0.991980645
TRINITY_DN95472_c1_g2(COMT)	TRINITY_DN83568_c0_g3(bHLH)	0.011575162	0.988424838
TRINITY_DN92541_c0_g1(CAD)	TRINITY_DN83645_c0_g5(G2-like)	0.017044087	0.982955913
TRINITY_DN87383_c2_g1(PAL)	TRINITY_DN83645_c0_g5(G2-like)	0.001644564	0.998355436
TRINITY_DN97112_c1_g2(HCT)	TRINITY_DN83645_c0_g5(G2-like)	0.002432968	0.997567032
TRINITY_DN95472_c1_g2(COMT)	TRINITY_DN83645_c0_g5(G2-like)	0.004555302	0.995444698
TRINITY_DN92541_c0_g1(CAD)	TRINITY_DN83873_c0_g1(AP2)	0.011410974	0.988589026
TRINITY_DN92541_c0_g1(CAD)	TRINITY_DN84051_c0_g1(C3H)	0.028910573	0.971089427
TRINITY_DN87383_c2_g1(PAL)	TRINITY_DN84051_c0_g1(C3H)	0.007322613	0.992677387
TRINITY_DN97112_c1_g2(HCT)	TRINITY_DN84051_c0_g1(C3H)	0.003343877	0.996656123
TRINITY_DN95472_c1_g2(COMT)	TRINITY_DN84051_c0_g1(C3H)	0.001658686	0.998341314
TRINITY_DN83539_c2_g6(C4H)	TRINITY_DN84051_c0_g1(C3H)	0.023529344	0.976470656
TRINITY_DN92541_c0_g1(CAD)	TRINITY_DN84540_c1_g3(WRKY)	0.038356908	-0.961643092
TRINITY_DN87383_c2_g1(PAL)	TRINITY_DN84540_c1_g3(WRKY)	0.027858401	-0.972141599
TRINITY_DN97112_c1_g2(HCT)	TRINITY_DN84540_c1_g3(WRKY)	0.035601204	-0.964398796
TRINITY_DN95472_c1_g2(COMT)	TRINITY_DN84540_c1_g3(WRKY)	0.042705745	-0.957294255
TRINITY_DN92541_c0_g1(CAD)	TRINITY_DN85014_c2_g5(ERF)	0.039335092	0.960664908
TRINITY_DN87383_c2_g1(PAL)	TRINITY_DN85014_c2_g5(ERF)	0.047872014	0.952127986
TRINITY_DN93270_c1_g1(UGT72E)	TRINITY_DN85072_c1_g2(MYB)	0.046908997	-0.953091003
TRINITY_DN92541_c0_g1(CAD)	TRINITY_DN85072_c1_g3(bHLH)	0.03413554	0.96586446

TRINITY_DN92541_c0_g1(CAD)	TRINITY_DN85120_c2_g1(NF-YA)	0.0024139	0.9975861
TRINITY_DN87383_c2_g1(PAL)	TRINITY_DN85120_c2_g1(NF-YA)	0.013788589	0.986211411
TRINITY_DN97112_c1_g2(HCT)	TRINITY_DN85120_c2_g1(NF-YA)	0.009370399	0.990629601
TRINITY_DN95472_c1_g2(COMT)	TRINITY_DN85120_c2_g1(NF-YA)	0.011033349	0.988966651
TRINITY_DN92541_c0_g1(CAD)	TRINITY_DN85212_c2_g4(HD-ZIP)	0.011010487	0.988989513
TRINITY_DN87383_c2_g1(PAL)	TRINITY_DN85212_c2_g4(HD-ZIP)	0.01693333	0.98306667
TRINITY_DN97112_c1_g2(HCT)	TRINITY_DN85212_c2_g4(HD-ZIP)	0.017423799	0.982576201
TRINITY_DN95472_c1_g2(COMT)	TRINITY_DN85212_c2_g4(HD-ZIP)	0.021666131	0.978333869
TRINITY_DN92541_c0_g1(CAD)	TRINITY_DN85243_c0_g4(ERF)	0.013259974	0.986740026
TRINITY_DN87383_c2_g1(PAL)	TRINITY_DN85243_c0_g4(ERF)	0.005568195	0.994431805
TRINITY_DN97112_c1_g2(HCT)	TRINITY_DN85243_c0_g4(ERF)	0.006418083	0.993581917
TRINITY_DN95472_c1_g2(COMT)	TRINITY_DN85243_c0_g4(ERF)	0.009422195	0.990577805
TRINITY_DN92541_c0_g1(CAD)	TRINITY_DN85339_c0_g4(MYB)	0.008823637	0.991176363
TRINITY_DN87383_c2_g1(PAL)	TRINITY_DN85339_c0_g4(MYB)	0.011445695	0.988554305
TRINITY_DN97112_c1_g2(HCT)	TRINITY_DN85339_c0_g4(MYB)	0.011094871	0.988905129
TRINITY_DN95472_c1_g2(COMT)	TRINITY_DN85339_c0_g4(MYB)	0.014441879	0.985558121
TRINITY_DN92541_c0_g1(CAD)	TRINITY_DN85820_c0_g4(HD-ZIP)	0.017208054	0.982791946
TRINITY_DN87383_c2_g1(PAL)	TRINITY_DN85820_c0_g4(HD-ZIP)	0.017276405	0.982723595
TRINITY_DN97112_c1_g2(HCT)	TRINITY_DN85820_c0_g4(HD-ZIP)	0.019915773	0.980084227
TRINITY_DN95472_c1_g2(COMT)	TRINITY_DN85820_c0_g4(HD-ZIP)	0.02490615	0.97509385
TRINITY_DN92541_c0_g1(CAD)	TRINITY_DN85820_c0_g5(HD-ZIP)	0.020751099	0.979248901
TRINITY_DN87383_c2_g1(PAL)	TRINITY_DN85820_c0_g5(HD-ZIP)	0.019200006	0.980799994
TRINITY_DN97112_c1_g2(HCT)	TRINITY_DN85820_c0_g5(HD-ZIP)	0.022836055	0.977163945
TRINITY_DN95472_c1_g2(COMT)	TRINITY_DN85820_c0_g5(HD-ZIP)	0.028289059	0.971710941
TRINITY_DN93270_c1_g1(UGT72E)	TRINITY_DN85951_c0_g1(GRF)	0.002467345	0.997532655
TRINITY_DN92541_c0_g1(CAD)	TRINITY_DN85971_c1_g1(ERF)	0.020543086	0.979456914
TRINITY_DN87383_c2_g1(PAL)	TRINITY_DN85971_c1_g1(ERF)	0.023342353	0.976657647
TRINITY_DN97112_c1_g2(HCT)	TRINITY_DN85971_c1_g1(ERF)	0.026651811	0.973348189
TRINITY_DN95472_c1_g2(COMT)	TRINITY_DN85971_c1_g1(ERF)	0.032360977	0.967639023
TRINITY_DN83539_c2_g6(C4H)	TRINITY_DN86187_c0_g1(G2-like)	0.037310826	-0.962689174
TRINITY_DN92541_c0_g1(CAD)	TRINITY_DN86251_c0_g1(LBD)	0.019817454	0.980182546
TRINITY_DN87383_c2_g1(PAL)	TRINITY_DN86251_c0_g1(LBD)	0.021520924	0.978479076
TRINITY_DN97112_c1_g2(HCT)	TRINITY_DN86251_c0_g1(LBD)	0.024739196	0.975260804
TRINITY_DN95472_c1_g2(COMT)	TRINITY_DN86251_c0_g1(LBD)	0.030273547	0.969726453
TRINITY_DN92541_c0_g1(CAD)	TRINITY_DN86264_c3_g2(ERF)	0.036397432	0.963602568
TRINITY_DN87383_c2_g1(PAL)	TRINITY_DN86264_c3_g2(ERF)	0.049959283	0.950040717
TRINITY_DN92541_c0_g1(CAD)	TRINITY_DN86264_c4_g1(ERF)	0.005054001	0.994945999
TRINITY_DN87383_c2_g1(PAL)	TRINITY_DN86264_c4_g1(ERF)	0.022155751	0.977844249
TRINITY_DN97112_c1_g2(HCT)	TRINITY_DN86264_c4_g1(ERF)	0.019580801	0.980419199
TRINITY_DN95472_c1_g2(COMT)	TRINITY_DN86264_c4_g1(ERF)	0.023050632	0.976949368
TRINITY_DN92541_c0_g1(CAD)	TRINITY_DN86264_c4_g2(ERF)	0.002125164	0.997874836
TRINITY_DN87383_c2_g1(PAL)	TRINITY_DN86264_c4_g2(ERF)	0.020197602	0.979802398

TRINITY_DN97112_c1_g2(HCT)	TRINITY_DN86264_c4_g2(ERF)	0.015920511	0.984079489
TRINITY_DN95472_c1_g2(COMT)	TRINITY_DN86264_c4_g2(ERF)	0.018396781	0.981603219
TRINITY_DN92541_c0_g1(CAD)	TRINITY_DN86340_c1_g1(ERF)	0.029524022	0.970475978
TRINITY_DN92541_c0_g1(CAD)	TRINITY_DN86562_c2_g4(bHLH)	0.016703539	0.983296461
TRINITY_DN87383_c2_g1(PAL)	TRINITY_DN86562_c2_g4(bHLH)	0.009530888	0.990469112
TRINITY_DN97112_c1_g2(HCT)	TRINITY_DN86562_c2_g4(bHLH)	0.012019991	0.987980009
TRINITY_DN95472_c1_g2(COMT)	TRINITY_DN86562_c2_g4(bHLH)	0.016135275	0.983864725
TRINITY_DN92541_c0_g1(CAD)	TRINITY_DN86602_c0_g2(WRKY)	0.030810395	0.969189605
TRINITY_DN87383_c2_g1(PAL)	TRINITY_DN86602_c0_g2(WRKY)	0.008092356	0.991907644
TRINITY_DN97112_c1_g2(HCT)	TRINITY_DN86602_c0_g2(WRKY)	0.014063564	0.985936436
TRINITY_DN95472_c1_g2(COMT)	TRINITY_DN86602_c0_g2(WRKY)	0.018725697	0.981274303
TRINITY_DN92541_c0_g1(CAD)	TRINITY_DN86766_c4_g1(CO-like)	0.039359449	0.960640551
TRINITY_DN92541_c0_g1(CAD)	TRINITY_DN86766_c4_g3(CO-like)	0.047900435	0.952099565
TRINITY_DN92541_c0_g1(CAD)	TRINITY_DN86884_c1_g7(MYB)	0.038416953	0.961583047
TRINITY_DN92541_c0_g1(CAD)	TRINITY_DN87187_c1_g1(GRAS)	0.015323011	0.984676989
TRINITY_DN87383_c2_g1(PAL)	TRINITY_DN87187_c1_g1(GRAS)	0.003293382	0.996706618
TRINITY_DN97112_c1_g2(HCT)	TRINITY_DN87187_c1_g1(GRAS)	0.004301738	0.995698262
TRINITY_DN95472_c1_g2(COMT)	TRINITY_DN87187_c1_g1(GRAS)	0.006935462	0.993064538
TRINITY_DN92541_c0_g1(CAD)	TRINITY_DN87293_c0_g1(NAC)	8.62E-04	0.999138015
TRINITY_DN87383_c2_g1(PAL)	TRINITY_DN87293_c0_g1(NAC)	0.018933742	0.981066258
TRINITY_DN97112_c1_g2(HCT)	TRINITY_DN87293_c0_g1(NAC)	0.011827628	0.988172372
TRINITY_DN95472_c1_g2(COMT)	TRINITY_DN87293_c0_g1(NAC)	0.012466956	0.987533044
TRINITY_DN92541_c0_g1(CAD)	TRINITY_DN87293_c0_g3(NAC)	0.001150331	0.998849669
TRINITY_DN87383_c2_g1(PAL)	TRINITY_DN87293_c0_g3(NAC)	0.018759644	0.981240356
TRINITY_DN97112_c1_g2(HCT)	TRINITY_DN87293_c0_g3(NAC)	0.011360133	0.988639867
TRINITY_DN95472_c1_g2(COMT)	TRINITY_DN87293_c0_g3(NAC)	0.011757817	0.988242183
TRINITY_DN83539_c2_g6(C4H)	TRINITY_DN87317_c2_g1(NAC)	0.018934768	-0.981065232
TRINITY_DN92541_c0_g1(CAD)	TRINITY_DN87469_c4_g1(Trihelix)	0.020124892	0.979875108
TRINITY_DN87383_c2_g1(PAL)	TRINITY_DN87469_c4_g1(Trihelix)	0.017365049	0.982634951
TRINITY_DN97112_c1_g2(HCT)	TRINITY_DN87469_c4_g1(Trihelix)	0.020895657	0.979104343
TRINITY_DN95472_c1_g2(COMT)	TRINITY_DN87469_c4_g1(Trihelix)	0.026152088	0.973847912
TRINITY_DN92541_c0_g1(CAD)	TRINITY_DN87586_c5_g1(MYB)	0.042679937	0.957320063
TRINITY_DN87383_c2_g1(PAL)	TRINITY_DN87586_c5_g1(MYB)	0.006228215	0.993771785
TRINITY_DN97112_c1_g2(HCT)	TRINITY_DN87586_c5_g1(MYB)	0.013764546	0.986235454
TRINITY_DN95472_c1_g2(COMT)	TRINITY_DN87586_c5_g1(MYB)	0.017928333	0.982071667
TRINITY_DN92541_c0_g1(CAD)	TRINITY_DN87612_c1_g3(TALE)	0.010790478	0.989209522
TRINITY_DN87383_c2_g1(PAL)	TRINITY_DN87612_c1_g3(TALE)	0.024418344	0.975581656
TRINITY_DN97112_c1_g2(HCT)	TRINITY_DN87612_c1_g3(TALE)	0.024230536	0.975769464
TRINITY_DN95472_c1_g2(COMT)	TRINITY_DN87612_c1_g3(TALE)	0.028861091	0.971138909
TRINITY_DN92541_c0_g1(CAD)	TRINITY_DN87710_c1_g2(CAMTA)	0.019817454	0.980182546
TRINITY_DN87383_c2_g1(PAL)	TRINITY_DN87710_c1_g2(CAMTA)	0.021520924	0.978479076
TRINITY_DN97112_c1_g2(HCT)	TRINITY_DN87710_c1_g2(CAMTA)	0.024739196	0.975260804

TRINITY_DN95472_c1_g2(COMT)	TRINITY_DN87710_c1_g2(CAMTA)	0.030273547	0.969726453
TRINITY_DN83539_c2_g6(C4H)	TRINITY_DN87748_c0_g1(FAR1)	0.032786131	0.967213869
TRINITY_DN92541_c0_g1(CAD)	TRINITY_DN88201_c1_g2(TALE)	0.031989772	0.968010228
TRINITY_DN92541_c0_g1(CAD)	TRINITY_DN88226_c2_g1(CO-like)	0.001244664	0.998755336
TRINITY_DN87383_c2_g1(PAL)	TRINITY_DN88226_c2_g1(CO-like)	0.038857342	0.961142658
TRINITY_DN97112_c1_g2(HCT)	TRINITY_DN88226_c2_g1(CO-like)	0.0283737	0.9716263
TRINITY_DN95472_c1_g2(COMT)	TRINITY_DN88226_c2_g1(CO-like)	0.028915927	0.971084073
TRINITY_DN92541_c0_g1(CAD)	TRINITY_DN88226_c2_g6(CO-like)	0.013325652	0.986674348
TRINITY_DN87383_c2_g1(PAL)	TRINITY_DN88226_c2_g6(CO-like)	0.020490029	0.979509971
TRINITY_DN97112_c1_g2(HCT)	TRINITY_DN88226_c2_g6(CO-like)	0.021625188	0.978374812
TRINITY_DN95472_c1_g2(COMT)	TRINITY_DN88226_c2_g6(CO-like)	0.026412942	0.973587058
TRINITY_DN92541_c0_g1(CAD)	TRINITY_DN88944_c2_g1(ERF)	0.022773393	0.977226607
TRINITY_DN87383_c2_g1(PAL)	TRINITY_DN88944_c2_g1(ERF)	0.018966124	0.981033876
TRINITY_DN97112_c1_g2(HCT)	TRINITY_DN88944_c2_g1(ERF)	0.023197428	0.976802572
TRINITY_DN95472_c1_g2(COMT)	TRINITY_DN88944_c2_g1(ERF)	0.028788376	0.971211624
TRINITY_DN92541_c0_g1(CAD)	TRINITY_DN88965_c0_g5(TALE)	3.55E-04	0.999645067
TRINITY_DN87383_c2_g1(PAL)	TRINITY_DN88965_c0_g5(TALE)	0.030501228	0.969498772
TRINITY_DN97112_c1_g2(HCT)	TRINITY_DN88965_c0_g5(TALE)	0.021065739	0.978934262
TRINITY_DN95472_c1_g2(COMT)	TRINITY_DN88965_c0_g5(TALE)	0.021489811	0.978510189
TRINITY_DN92541_c0_g1(CAD)	TRINITY_DN89286_c0_g5(CO-like)	0.027047779	0.972952221
TRINITY_DN87383_c2_g1(PAL)	TRINITY_DN89286_c0_g5(CO-like)	0.031221285	0.968778715
TRINITY_DN97112_c1_g2(HCT)	TRINITY_DN89286_c0_g5(CO-like)	0.035758288	0.964241712
TRINITY_DN95472_c1_g2(COMT)	TRINITY_DN89286_c0_g5(CO-like)	0.04239254	0.95760746
TRINITY_DN92541_c0_g1(CAD)	TRINITY_DN89343_c1_g1(ARR-B)	0.017381487	0.982618513
TRINITY_DN87383_c2_g1(PAL)	TRINITY_DN89343_c1_g1(ARR-B)	0.001592274	0.998407726
TRINITY_DN97112_c1_g2(HCT)	TRINITY_DN89343_c1_g1(ARR-B)	0.002490196	0.997509804
TRINITY_DN95472_c1_g2(COMT)	TRINITY_DN89343_c1_g1(ARR-B)	0.004640759	0.995359241
TRINITY_DN92541_c0_g1(CAD)	TRINITY_DN89360_c1_g9(MYB)	0.001278476	0.998721524
TRINITY_DN87383_c2_g1(PAL)	TRINITY_DN89360_c1_g9(MYB)	0.032792825	0.967207175
TRINITY_DN97112_c1_g2(HCT)	TRINITY_DN89360_c1_g9(MYB)	0.02216423	0.97783577
TRINITY_DN95472_c1_g2(COMT)	TRINITY_DN89360_c1_g9(MYB)	0.021955961	0.978044039
TRINITY_DN87383_c2_g1(PAL)	TRINITY_DN89381_c3_g2(NF-YA)	0.043073378	0.956926622
TRINITY_DN97112_c1_g2(HCT)	TRINITY_DN89381_c3_g2(NF-YA)	0.034530052	0.965469948
TRINITY_DN95472_c1_g2(COMT)	TRINITY_DN89381_c3_g2(NF-YA)	0.028487198	0.971512802
TRINITY_DN83539_c2_g6(C4H)	TRINITY_DN89381_c3_g2(NF-YA)	0.00113799	0.99886201
TRINITY_DN87383_c2_g1(PAL)	TRINITY_DN89381_c3_g4(WOX)	0.042230086	0.957769914
TRINITY_DN97112_c1_g2(HCT)	TRINITY_DN89381_c3_g4(WOX)	0.031301966	0.968698034
TRINITY_DN95472_c1_g2(COMT)	TRINITY_DN89381_c3_g4(WOX)	0.025220966	0.974779034
TRINITY_DN83539_c2_g6(C4H)	TRINITY_DN89381_c3_g4(WOX)	0.005497243	0.994502757
TRINITY_DN92541_c0_g1(CAD)	TRINITY_DN89502_c0_g1(C2H2)	0.011202385	0.988797615
TRINITY_DN97112_c1_g2(HCT)	TRINITY_DN89502_c0_g1(C2H2)	0.041744167	0.958255833
TRINITY_DN95472_c1_g2(COMT)	TRINITY_DN89502_c0_g1(C2H2)	0.039477729	0.960522271

TRINITY_DN92541_c0_g1(CAD)	TRINITY_DN89502_c0_g3(C2H2)	0.018667858	0.981332142
TRINITY_DN87383_c2_g1(PAL)	TRINITY_DN89502_c0_g3(C2H2)	0.030429716	0.969570284
TRINITY_DN97112_c1_g2(HCT)	TRINITY_DN89502_c0_g3(C2H2)	0.032440144	0.967559856
TRINITY_DN95472_c1_g2(COMT)	TRINITY_DN89502_c0_g3(C2H2)	0.038274347	0.961725653
TRINITY_DN92541_c0_g1(CAD)	TRINITY_DN89624_c1_g1(NAC)	0.015316953	0.984683047
TRINITY_DN87383_c2_g1(PAL)	TRINITY_DN89624_c1_g1(NAC)	0.019793476	0.980206524
TRINITY_DN97112_c1_g2(HCT)	TRINITY_DN89624_c1_g1(NAC)	0.021675214	0.978324786
TRINITY_DN95472_c1_g2(COMT)	TRINITY_DN89624_c1_g1(NAC)	0.026653052	0.973346948
TRINITY_DN92541_c0_g1(CAD)	TRINITY_DN89664_c3_g1(bHLH)	0.005913176	0.994086824
TRINITY_DN87383_c2_g1(PAL)	TRINITY_DN89664_c3_g1(bHLH)	0.035358198	0.964641802
TRINITY_DN97112_c1_g2(HCT)	TRINITY_DN89664_c3_g1(bHLH)	0.02264979	0.97735021
TRINITY_DN95472_c1_g2(COMT)	TRINITY_DN89664_c3_g1(bHLH)	0.020850008	0.979149992
TRINITY_DN92541_c0_g1(CAD)	TRINITY_DN89714_c2_g2(bHLH)	0.006456401	0.993543599
TRINITY_DN87383_c2_g1(PAL)	TRINITY_DN89714_c2_g2(bHLH)	0.014508775	0.985491225
TRINITY_DN97112_c1_g2(HCT)	TRINITY_DN89714_c2_g2(bHLH)	0.013087818	0.986912182
TRINITY_DN95472_c1_g2(COMT)	TRINITY_DN89714_c2_g2(bHLH)	0.016330459	0.983669541
TRINITY_DN92541_c0_g1(CAD)	TRINITY_DN89720_c1_g3(NAC)	0.009032787	0.990967213
TRINITY_DN87383_c2_g1(PAL)	TRINITY_DN89720_c1_g3(NAC)	0.019586636	0.980413364
TRINITY_DN97112_c1_g2(HCT)	TRINITY_DN89720_c1_g3(NAC)	0.019116848	0.980883152
TRINITY_DN95472_c1_g2(COMT)	TRINITY_DN89720_c1_g3(NAC)	0.023239037	0.976760963
TRINITY_DN92541_c0_g1(CAD)	TRINITY_DN89720_c1_g4(NAC)	0.013332677	0.986667323
TRINITY_DN87383_c2_g1(PAL)	TRINITY_DN89720_c1_g4(NAC)	0.016197358	0.983802642
TRINITY_DN97112_c1_g2(HCT)	TRINITY_DN89720_c1_g4(NAC)	0.017579988	0.982420012
TRINITY_DN95472_c1_g2(COMT)	TRINITY_DN89720_c1_g4(NAC)	0.02206621	0.97793379
TRINITY_DN92541_c0_g1(CAD)	TRINITY_DN89720_c1_g5(NAC)	0.031717462	0.968282538
TRINITY_DN87383_c2_g1(PAL)	TRINITY_DN89720_c1_g5(NAC)	0.044685391	0.955314609
TRINITY_DN97112_c1_g2(HCT)	TRINITY_DN89720_c1_g5(NAC)	0.048980015	0.951019985
TRINITY_DN92541_c0_g1(CAD)	TRINITY_DN89720_c1_g7(NAC)	0.018618575	0.981381425
TRINITY_DN87383_c2_g1(PAL)	TRINITY_DN89720_c1_g7(NAC)	0.021385203	0.978614797
TRINITY_DN97112_c1_g2(HCT)	TRINITY_DN89720_c1_g7(NAC)	0.024239405	0.975760595
TRINITY_DN95472_c1_g2(COMT)	TRINITY_DN89720_c1_g7(NAC)	0.029655183	0.970344817
TRINITY_DN92541_c0_g1(CAD)	TRINITY_DN90039_c0_g3(TCP)	0.011427733	-0.988572267
TRINITY_DN87383_c2_g1(PAL)	TRINITY_DN90039_c0_g3(TCP)	0.046858637	-0.953141363
TRINITY_DN97112_c1_g2(HCT)	TRINITY_DN90039_c0_g3(TCP)	0.043188497	-0.956811503
TRINITY_DN95472_c1_g2(COMT)	TRINITY_DN90039_c0_g3(TCP)	0.047896259	-0.952103741
TRINITY_DN92541_c0_g1(CAD)	TRINITY_DN90067_c0_g3(C3H)	0.024309737	0.975690263
TRINITY_DN87383_c2_g1(PAL)	TRINITY_DN90067_c0_g3(C3H)	0.014367051	0.985632949
TRINITY_DN97112_c1_g2(HCT)	TRINITY_DN90067_c0_g3(C3H)	0.00693702	0.99306298
TRINITY_DN95472_c1_g2(COMT)	TRINITY_DN90067_c0_g3(C3H)	0.004273875	0.995726125
TRINITY_DN83539_c2_g6(C4H)	TRINITY_DN90067_c0_g3(C3H)	0.023429604	0.976570396
TRINITY_DN92541_c0_g1(CAD)	TRINITY_DN90195_c1_g2(ERF)	0.04203022	-0.95796978
TRINITY_DN83539_c2_g6(C4H)	TRINITY_DN90205_c1_g1(GRAS)	0.047808903	-0.952191097

TRINITY_DN92541_c0_g1(CAD)	TRINITY_DN90479_c0_g6(Trihelix)	0.01661759	0.98338241
TRINITY_DN87383_c2_g1(PAL)	TRINITY_DN90479_c0_g6(Trihelix)	0.021242012	0.978757988
TRINITY_DN97112_c1_g2(HCT)	TRINITY_DN90479_c0_g6(Trihelix)	0.023459661	0.976540339
TRINITY_DN95472_c1_g2(COMT)	TRINITY_DN90479_c0_g6(Trihelix)	0.028667693	0.971332307
TRINITY_DN92541_c0_g1(CAD)	TRINITY_DN90865_c3_g4(HD-ZIP)	0.03100991	0.96899009
TRINITY_DN87383_c2_g1(PAL)	TRINITY_DN90865_c3_g4(HD-ZIP)	0.026062588	0.973937412
TRINITY_DN97112_c1_g2(HCT)	TRINITY_DN90865_c3_g4(HD-ZIP)	0.015600417	0.984399583
TRINITY_DN95472_c1_g2(COMT)	TRINITY_DN90865_c3_g4(HD-ZIP)	0.011479155	0.988520845
TRINITY_DN83539_c2_g6(C4H)	TRINITY_DN90865_c3_g4(HD-ZIP)	0.019319806	0.980680194
TRINITY_DN83539_c2_g6(C4H)	TRINITY_DN90873_c0_g1(GRAS)	0.019368044	-0.980631956
TRINITY_DN93270_c1_g1(UGT72E)	TRINITY_DN90873_c0_g4(GRAS)	0.017473612	0.982526388
TRINITY_DN92541_c0_g1(CAD)	TRINITY_DN90941_c3_g1(ERF)	0.02205781	0.97794219
TRINITY_DN92541_c0_g1(CAD)	TRINITY_DN91013_c3_g1(LBD)	0.019817454	0.980182546
TRINITY_DN87383_c2_g1(PAL)	TRINITY_DN91013_c3_g1(LBD)	0.021520924	0.978479076
TRINITY_DN97112_c1_g2(HCT)	TRINITY_DN91013_c3_g1(LBD)	0.024739196	0.975260804
TRINITY_DN95472_c1_g2(COMT)	TRINITY_DN91013_c3_g1(LBD)	0.030273547	0.969726453
TRINITY_DN92541_c0_g1(CAD)	TRINITY_DN91170_c2_g1(bHLH)	0.033982796	0.966017204
TRINITY_DN87383_c2_g1(PAL)	TRINITY_DN91170_c2_g1(bHLH)	0.042775059	0.957224941
TRINITY_DN97112_c1_g2(HCT)	TRINITY_DN91170_c2_g1(bHLH)	0.047986876	0.952013124
TRINITY_DN92541_c0_g1(CAD)	TRINITY_DN91170_c2_g3(bHLH)	0.005992578	0.994007422
TRINITY_DN87383_c2_g1(PAL)	TRINITY_DN91170_c2_g3(bHLH)	0.036488538	0.963511462
TRINITY_DN97112_c1_g2(HCT)	TRINITY_DN91170_c2_g3(bHLH)	0.032184325	0.967815675
TRINITY_DN95472_c1_g2(COMT)	TRINITY_DN91170_c2_g3(bHLH)	0.035966606	0.964033394
TRINITY_DN87383_c2_g1(PAL)	TRINITY_DN91240_c0_g2(G2-like)	0.010182406	0.989817594
TRINITY_DN97112_c1_g2(HCT)	TRINITY_DN91240_c0_g2(G2-like)	0.012918429	0.987081571
TRINITY_DN95472_c1_g2(COMT)	TRINITY_DN91240_c0_g2(G2-like)	0.012222967	0.987777033
TRINITY_DN83539_c2_g6(C4H)	TRINITY_DN91240_c0_g2(G2-like)	0.025493255	0.974506745
TRINITY_DN87383_c2_g1(PAL)	TRINITY_DN91261_c1_g1(bZIP)	0.040494099	-0.959505901
TRINITY_DN92541_c0_g1(CAD)	TRINITY_DN91398_c3_g4(C2H2)	0.018405974	0.981594026
TRINITY_DN87383_c2_g1(PAL)	TRINITY_DN91398_c3_g4(C2H2)	0.02136484	0.97863516
TRINITY_DN97112_c1_g2(HCT)	TRINITY_DN91398_c3_g4(C2H2)	0.024153196	0.975846804
TRINITY_DN95472_c1_g2(COMT)	TRINITY_DN91398_c3_g4(C2H2)	0.02954751	0.97045249
TRINITY_DN92541_c0_g1(CAD)	TRINITY_DN91488_c0_g3(CO-like)	0.014506086	0.985493914
TRINITY_DN87383_c2_g1(PAL)	TRINITY_DN91488_c0_g3(CO-like)	0.024819442	0.975180558
TRINITY_DN97112_c1_g2(HCT)	TRINITY_DN91488_c0_g3(CO-like)	0.025999505	0.974000495
TRINITY_DN95472_c1_g2(COMT)	TRINITY_DN91488_c0_g3(CO-like)	0.031151323	0.968848677
TRINITY_DN92541_c0_g1(CAD)	TRINITY_DN91612_c0_g1(ERF)	0.023961747	0.976038253
TRINITY_DN87383_c2_g1(PAL)	TRINITY_DN91612_c0_g1(ERF)	0.025748199	0.974251801
TRINITY_DN97112_c1_g2(HCT)	TRINITY_DN91612_c0_g1(ERF)	0.029888637	0.970111363
TRINITY_DN95472_c1_g2(COMT)	TRINITY_DN91612_c0_g1(ERF)	0.036018271	0.963981729
TRINITY_DN92541_c0_g1(CAD)	TRINITY_DN91730_c2_g1(MYB)	0.008856649	0.991143351
TRINITY_DN87383_c2_g1(PAL)	TRINITY_DN91730_c2_g1(MYB)	0.042086226	0.957913774

TRINITY_DN97112_c1_g2(HCT)	TRINITY_DN91730_c2_g1(MYB)	0.02785103	0.97214897
TRINITY_DN95472_c1_g2(COMT)	TRINITY_DN91730_c2_g1(MYB)	0.025455006	0.974544994
TRINITY_DN92541_c0_g1(CAD)	TRINITY_DN91736_c0_g1(ERF)	0.005335827	0.994664173
TRINITY_DN87383_c2_g1(PAL)	TRINITY_DN91736_c0_g1(ERF)	0.046967719	0.953032282
TRINITY_DN97112_c1_g2(HCT)	TRINITY_DN91736_c0_g1(ERF)	0.039550887	0.960449113
TRINITY_DN95472_c1_g2(COMT)	TRINITY_DN91736_c0_g1(ERF)	0.042507719	0.957492281
TRINITY_DN93270_c1_g1(UGT72E)	TRINITY_DN91739_c0_g1(LSD)	0.020154607	0.979845393
TRINITY_DN92541_c0_g1(CAD)	TRINITY_DN91892_c3_g1(bHLH)	0.02326985	0.97673015
TRINITY_DN87383_c2_g1(PAL)	TRINITY_DN91970_c1_g5(GRAS)	0.006392788	0.993607212
TRINITY_DN97112_c1_g2(HCT)	TRINITY_DN91970_c1_g5(GRAS)	0.01453995	0.98546005
TRINITY_DN95472_c1_g2(COMT)	TRINITY_DN91970_c1_g5(GRAS)	0.018220282	0.981779718
TRINITY_DN87383_c2_g1(PAL)	TRINITY_DN91970_c1_g6(GRAS)	0.031305843	0.968694157
TRINITY_DN97112_c1_g2(HCT)	TRINITY_DN91970_c1_g6(GRAS)	0.037912776	0.962087224
TRINITY_DN95472_c1_g2(COMT)	TRINITY_DN91970_c1_g6(GRAS)	0.036870042	0.963129958
TRINITY_DN83539_c2_g6(C4H)	TRINITY_DN91970_c1_g6(GRAS)	0.031132399	0.968867601
TRINITY_DN92541_c0_g1(CAD)	TRINITY_DN92066_c0_g4(CAMTA)	0.017984068	0.982015932
TRINITY_DN87383_c2_g1(PAL)	TRINITY_DN92066_c0_g4(CAMTA)	0.003818156	0.996181844
TRINITY_DN97112_c1_g2(HCT)	TRINITY_DN92066_c0_g4(CAMTA)	0.005936453	0.994063547
TRINITY_DN95472_c1_g2(COMT)	TRINITY_DN92066_c0_g4(CAMTA)	0.009053842	0.990946158
TRINITY_DN92541_c0_g1(CAD)	TRINITY_DN92202_c0_g4(MYB)	0.024949969	0.975050031
TRINITY_DN87383_c2_g1(PAL)	TRINITY_DN92202_c0_g4(MYB)	0.03444273	0.96555727
TRINITY_DN97112_c1_g2(HCT)	TRINITY_DN92202_c0_g4(MYB)	0.038001018	0.961998982
TRINITY_DN95472_c1_g2(COMT)	TRINITY_DN92202_c0_g4(MYB)	0.044577555	0.955422445
TRINITY_DN92541_c0_g1(CAD)	TRINITY_DN92454_c0_g4(TCP)	0.001892147	0.998107853
TRINITY_DN87383_c2_g1(PAL)	TRINITY_DN92454_c0_g4(TCP)	0.015629515	0.984370485
TRINITY_DN97112_c1_g2(HCT)	TRINITY_DN92454_c0_g4(TCP)	0.009020236	0.990979764
TRINITY_DN95472_c1_g2(COMT)	TRINITY_DN92454_c0_g4(TCP)	0.009513181	0.990486819
TRINITY_DN92541_c0_g1(CAD)	TRINITY_DN92660_c1_g6(ERF)	0.004877755	0.995122245
TRINITY_DN97112_c1_g2(HCT)	TRINITY_DN92660_c1_g6(ERF)	0.042588449	0.957411551
TRINITY_DN95472_c1_g2(COMT)	TRINITY_DN92660_c1_g6(ERF)	0.043942074	0.956057926
TRINITY_DN83539_c2_g6(C4H)	TRINITY_DN92695_c1_g4(MYB)	0.025459911	0.974540089
TRINITY_DN83539_c2_g6(C4H)	TRINITY_DN92774_c0_g3(MYB)	0.042460959	0.957539041
TRINITY_DN92541_c0_g1(CAD)	TRINITY_DN92856_c1_g1(C2H2)	0.022421059	0.977578941
TRINITY_DN92541_c0_g1(CAD)	TRINITY_DN92856_c1_g3(C2H2)	0.045924869	0.954075131
TRINITY_DN92541_c0_g1(CAD)	TRINITY_DN93054_c1_g2(ERF)	0.0022453	0.9977547
TRINITY_DN87383_c2_g1(PAL)	TRINITY_DN93054_c1_g2(ERF)	0.02379286	0.97620714
TRINITY_DN97112_c1_g2(HCT)	TRINITY_DN93054_c1_g2(ERF)	0.01928784	0.98071216
TRINITY_DN95472_c1_g2(COMT)	TRINITY_DN93054_c1_g2(ERF)	0.02198923	0.97801077
TRINITY_DN92541_c0_g1(CAD)	TRINITY_DN93097_c1_g5(C2H2)	0.012655326	0.987344674
TRINITY_DN97112_c1_g2(HCT)	TRINITY_DN93097_c1_g5(C2H2)	0.048391972	0.951608028
TRINITY_DN95472_c1_g2(COMT)	TRINITY_DN93097_c1_g5(C2H2)	0.046335866	0.953664134
TRINITY_DN92541_c0_g1(CAD)	TRINITY_DN93312_c3_g3(ERF)	0.026691463	0.973308537

TRINITY_DN87383_c2_g1(PAL)	TRINITY_DN93312_c3_g3(ERF)	0.038076087	0.961923913
TRINITY_DN97112_c1_g2(HCT)	TRINITY_DN93312_c3_g3(ERF)	0.041722614	0.958277386
TRINITY_DN95472_c1_g2(COMT)	TRINITY_DN93312_c3_g3(ERF)	0.048554313	0.951445687
TRINITY_DN92541_c0_g1(CAD)	TRINITY_DN93312_c3_g6(ERF)	0.021101133	0.978898867
TRINITY_DN87383_c2_g1(PAL)	TRINITY_DN93312_c3_g6(ERF)	0.026281097	0.973718903
TRINITY_DN97112_c1_g2(HCT)	TRINITY_DN93312_c3_g6(ERF)	0.029514755	0.970485245
TRINITY_DN95472_c1_g2(COMT)	TRINITY_DN93312_c3_g6(ERF)	0.03542885	0.96457115
TRINITY_DN92541_c0_g1(CAD)	TRINITY_DN93638_c2_g1(GATA)	6.94E-04	0.999306324
TRINITY_DN87383_c2_g1(PAL)	TRINITY_DN93638_c2_g1(GATA)	0.029218065	0.970781935
TRINITY_DN97112_c1_g2(HCT)	TRINITY_DN93638_c2_g1(GATA)	0.022399201	0.977600799
TRINITY_DN95472_c1_g2(COMT)	TRINITY_DN93638_c2_g1(GATA)	0.024350509	0.975649491
TRINITY_DN92541_c0_g1(CAD)	TRINITY_DN93638_c2_g3(GATA)	0.014752653	0.985247347
TRINITY_DN87383_c2_g1(PAL)	TRINITY_DN93638_c2_g3(GATA)	0.006231707	0.993768293
TRINITY_DN97112_c1_g2(HCT)	TRINITY_DN93638_c2_g3(GATA)	0.00775907	0.99224093
TRINITY_DN95472_c1_g2(COMT)	TRINITY_DN93638_c2_g3(GATA)	0.011109907	0.988890093
TRINITY_DN92541_c0_g1(CAD)	TRINITY_DN93638_c2_g5(GATA)	0.024839186	0.975160814
TRINITY_DN87383_c2_g1(PAL)	TRINITY_DN93638_c2_g5(GATA)	0.019064733	0.980935267
TRINITY_DN97112_c1_g2(HCT)	TRINITY_DN93638_c2_g5(GATA)	0.023863088	0.976136912
TRINITY_DN95472_c1_g2(COMT)	TRINITY_DN93638_c2_g5(GATA)	0.029604568	0.970395432
TRINITY_DN92541_c0_g1(CAD)	TRINITY_DN93835_c2_g3(GRAS)	0.009776236	0.990223764
TRINITY_DN87383_c2_g1(PAL)	TRINITY_DN93835_c2_g3(GRAS)	0.013098904	0.986901096
TRINITY_DN97112_c1_g2(HCT)	TRINITY_DN93835_c2_g3(GRAS)	0.013190826	0.986809174
TRINITY_DN95472_c1_g2(COMT)	TRINITY_DN93835_c2_g3(GRAS)	0.016898169	0.983101831
TRINITY_DN92541_c0_g1(CAD)	TRINITY_DN94086_c1_g4(ERF)	0.015097866	0.984902134
TRINITY_DN87383_c2_g1(PAL)	TRINITY_DN94086_c1_g4(ERF)	0.003331565	0.996668435
TRINITY_DN97112_c1_g2(HCT)	TRINITY_DN94086_c1_g4(ERF)	0.004264182	0.995735818
TRINITY_DN95472_c1_g2(COMT)	TRINITY_DN94086_c1_g4(ERF)	0.006877692	0.993122308
TRINITY_DN92541_c0_g1(CAD)	TRINITY_DN94332_c1_g4(MYB)	0.004725489	0.995274511
TRINITY_DN87383_c2_g1(PAL)	TRINITY_DN94332_c1_g4(MYB)	0.014106407	0.985893593
TRINITY_DN97112_c1_g2(HCT)	TRINITY_DN94332_c1_g4(MYB)	0.011719521	0.988280479
TRINITY_DN95472_c1_g2(COMT)	TRINITY_DN94332_c1_g4(MYB)	0.014482231	0.985517769
TRINITY_DN92541_c0_g1(CAD)	TRINITY_DN94512_c1_g4(ERF)	0.023837055	0.976162945
TRINITY_DN87383_c2_g1(PAL)	TRINITY_DN94512_c1_g4(ERF)	0.017060691	0.982939309
TRINITY_DN97112_c1_g2(HCT)	TRINITY_DN94512_c1_g4(ERF)	0.021653124	0.978346876
TRINITY_DN95472_c1_g2(COMT)	TRINITY_DN94512_c1_g4(ERF)	0.027156141	0.972843859
TRINITY_DN92541_c0_g1(CAD)	TRINITY_DN94512_c1_g5(C2H2)	0.014492593	0.985507407
TRINITY_DN87383_c2_g1(PAL)	TRINITY_DN94512_c1_g5(C2H2)	0.026117997	0.973882003
TRINITY_DN97112_c1_g2(HCT)	TRINITY_DN94512_c1_g5(C2H2)	0.027158416	0.972841584
TRINITY_DN95472_c1_g2(COMT)	TRINITY_DN94512_c1_g5(C2H2)	0.03236055	0.96763945
TRINITY_DN92541_c0_g1(CAD)	TRINITY_DN94789_c0_g2(MYB)	0.013171159	0.986828841
TRINITY_DN87383_c2_g1(PAL)	TRINITY_DN94789_c0_g2(MYB)	0.015260241	0.984739759
TRINITY_DN97112_c1_g2(HCT)	TRINITY_DN94789_c0_g2(MYB)	0.016616605	0.983383395

TRINITY_DN95472_c1_g2(COMT)	TRINITY_DN94789_c0_g2(MYB)	0.021002832	0.978997168
TRINITY_DN92541_c0_g1(CAD)	TRINITY_DN94853_c2_g3(TALE)	0.039680423	0.960319577
TRINITY_DN83539_c2_g6(C4H)	TRINITY_DN95186_c1_g3(CAMTA)	0.031140474	0.968859526
TRINITY_DN83539_c2_g6(C4H)	TRINITY_DN95706_c2_g5(C3H)	0.042182652	0.957817348
TRINITY_DN97112_c1_g2(HCT)	TRINITY_DN95706_c2_g7(C3H)	0.048319455	0.951680545
TRINITY_DN95472_c1_g2(COMT)	TRINITY_DN95706_c2_g7(C3H)	0.042958275	0.957041725
TRINITY_DN83539_c2_g6(C4H)	TRINITY_DN95706_c2_g7(C3H)	0.004446573	0.995553427
TRINITY_DN92541_c0_g1(CAD)	TRINITY_DN95793_c0_g3(WRKY)	0.027264708	0.972735292
TRINITY_DN87383_c2_g1(PAL)	TRINITY_DN95793_c0_g3(WRKY)	0.005950247	0.994049753
TRINITY_DN97112_c1_g2(HCT)	TRINITY_DN95793_c0_g3(WRKY)	0.010888416	0.989111584
TRINITY_DN95472_c1_g2(COMT)	TRINITY_DN95793_c0_g3(WRKY)	0.015041047	0.984958953
TRINITY_DN92541_c0_g1(CAD)	TRINITY_DN95793_c0_g5(WRKY)	0.021113242	0.978886758
TRINITY_DN87383_c2_g1(PAL)	TRINITY_DN95793_c0_g5(WRKY)	0.005791746	0.994208254
TRINITY_DN97112_c1_g2(HCT)	TRINITY_DN95793_c0_g5(WRKY)	0.009210815	0.990789185
TRINITY_DN95472_c1_g2(COMT)	TRINITY_DN95793_c0_g5(WRKY)	0.013040003	0.986959997
TRINITY_DN92541_c0_g1(CAD)	TRINITY_DN95793_c0_g6(WRKY)	0.027462806	0.972537194
TRINITY_DN87383_c2_g1(PAL)	TRINITY_DN95793_c0_g6(WRKY)	0.001079584	0.998920416
TRINITY_DN97112_c1_g2(HCT)	TRINITY_DN95793_c0_g6(WRKY)	0.004530684	0.995469316
TRINITY_DN95472_c1_g2(COMT)	TRINITY_DN95793_c0_g6(WRKY)	0.007169611	0.992830389
TRINITY_DN92541_c0_g1(CAD)	TRINITY_DN96274_c0_g3(C3H)	0.031692814	0.968307186
TRINITY_DN87383_c2_g1(PAL)	TRINITY_DN96274_c0_g3(C3H)	0.001906143	0.998093857
TRINITY_DN97112_c1_g2(HCT)	TRINITY_DN96274_c0_g3(C3H)	0.006525475	0.993474525
TRINITY_DN95472_c1_g2(COMT)	TRINITY_DN96274_c0_g3(C3H)	0.009544468	0.990455532
TRINITY_DN92541_c0_g1(CAD)	TRINITY_DN96383_c0_g1(TALE)	0.00308666	0.99691334
TRINITY_DN87383_c2_g1(PAL)	TRINITY_DN96383_c0_g1(TALE)	0.012145227	0.987854773
TRINITY_DN97112_c1_g2(HCT)	TRINITY_DN96383_c0_g1(TALE)	0.006663936	0.993336064
TRINITY_DN95472_c1_g2(COMT)	TRINITY_DN96383_c0_g1(TALE)	0.00738364	0.99261636
TRINITY_DN92541_c0_g1(CAD)	TRINITY_DN96383_c0_g11(TALE)	0.003343608	0.996656392
TRINITY_DN87383_c2_g1(PAL)	TRINITY_DN96383_c0_g11(TALE)	0.01504909	0.98495091
TRINITY_DN97112_c1_g2(HCT)	TRINITY_DN96383_c0_g11(TALE)	0.011757374	0.988242626
TRINITY_DN95472_c1_g2(COMT)	TRINITY_DN96383_c0_g11(TALE)	0.014185907	0.985814093
TRINITY_DN92541_c0_g1(CAD)	TRINITY_DN96383_c0_g4(TALE)	0.013777422	0.986222578
TRINITY_DN87383_c2_g1(PAL)	TRINITY_DN96383_c0_g4(TALE)	0.021297415	0.978702585
TRINITY_DN97112_c1_g2(HCT)	TRINITY_DN96383_c0_g4(TALE)	0.022533186	0.977466814
TRINITY_DN95472_c1_g2(COMT)	TRINITY_DN96383_c0_g4(TALE)	0.027423172	0.972576828
TRINITY_DN92541_c0_g1(CAD)	TRINITY_DN96468_c3_g2(TALE)	0.035894742	0.964105258
TRINITY_DN92541_c0_g1(CAD)	TRINITY_DN96468_c3_g4(TALE)	0.048297812	0.951702188
TRINITY_DN92541_c0_g1(CAD)	TRINITY_DN96756_c1_g2(HSF)	0.016270762	0.983729238
TRINITY_DN87383_c2_g1(PAL)	TRINITY_DN96756_c1_g2(HSF)	0.019872114	0.980127886
TRINITY_DN97112_c1_g2(HCT)	TRINITY_DN96756_c1_g2(HSF)	0.022068758	0.977931242
TRINITY_DN95472_c1_g2(COMT)	TRINITY_DN96756_c1_g2(HSF)	0.027154211	0.972845789
TRINITY_DN92541_c0_g1(CAD)	TRINITY_DN96881_c3_g4(TCP)	0.028652112	-0.971347888

TRINITY_DN92541_c0_g1(CAD)	TRINITY_DN97070_c1_g1(ERF)	0.049473659	0.950526341
TRINITY_DN87383_c2_g1(PAL)	TRINITY_DN97070_c1_g1(ERF)	0.03620045	0.963799551
TRINITY_DN97112_c1_g2(HCT)	TRINITY_DN97070_c1_g1(ERF)	0.045927545	0.954072455
TRINITY_DN92541_c0_g1(CAD)	TRINITY_DN97140_c3_g1(bHLH)	0.002636291	0.997363709
TRINITY_DN87383_c2_g1(PAL)	TRINITY_DN97140_c3_g1(bHLH)	0.021295371	0.978704629
TRINITY_DN97112_c1_g2(HCT)	TRINITY_DN97140_c3_g1(bHLH)	0.012257876	0.987742124
TRINITY_DN95472_c1_g2(COMT)	TRINITY_DN97140_c3_g1(bHLH)	0.011697591	0.988302409
TRINITY_DN92541_c0_g1(CAD)	TRINITY_DN97293_c2_g1(CO-like)	0.002479215	0.997520785
TRINITY_DN87383_c2_g1(PAL)	TRINITY_DN97293_c2_g1(CO-like)	0.025870412	0.974129588
TRINITY_DN97112_c1_g2(HCT)	TRINITY_DN97293_c2_g1(CO-like)	0.021266896	0.978733104
TRINITY_DN95472_c1_g2(COMT)	TRINITY_DN97293_c2_g1(CO-like)	0.024101748	0.975898252
TRINITY_DN92541_c0_g1(CAD)	TRINITY_DN97293_c2_g10(CO-like)	0.010972217	0.989027783
TRINITY_DN87383_c2_g1(PAL)	TRINITY_DN97293_c2_g10(CO-like)	0.0205154	0.9794846
TRINITY_DN97112_c1_g2(HCT)	TRINITY_DN97293_c2_g10(CO-like)	0.020763432	0.979236568
TRINITY_DN95472_c1_g2(COMT)	TRINITY_DN97293_c2_g10(CO-like)	0.025237591	0.974762409
TRINITY_DN92541_c0_g1(CAD)	TRINITY_DN97293_c2_g11(CO-like)	0.018465973	0.981534027
TRINITY_DN87383_c2_g1(PAL)	TRINITY_DN97293_c2_g11(CO-like)	0.021772208	0.978227792
TRINITY_DN97112_c1_g2(HCT)	TRINITY_DN97293_c2_g11(CO-like)	0.024550717	0.975449283
TRINITY_DN95472_c1_g2(COMT)	TRINITY_DN97293_c2_g11(CO-like)	0.029975793	0.970024207
TRINITY_DN92541_c0_g1(CAD)	TRINITY_DN97293_c2_g6(CO-like)	0.006869708	0.993130292
TRINITY_DN87383_c2_g1(PAL)	TRINITY_DN97293_c2_g6(CO-like)	0.023041178	0.976958822
TRINITY_DN97112_c1_g2(HCT)	TRINITY_DN97293_c2_g6(CO-like)	0.021291908	0.978708092
TRINITY_DN95472_c1_g2(COMT)	TRINITY_DN97293_c2_g6(CO-like)	0.025192963	0.974807037
TRINITY_DN92541_c0_g1(CAD)	TRINITY_DN97293_c2_g7(CO-like)	0.010898026	0.989101974
TRINITY_DN87383_c2_g1(PAL)	TRINITY_DN97293_c2_g7(CO-like)	0.018916439	0.981083561
TRINITY_DN97112_c1_g2(HCT)	TRINITY_DN97293_c2_g7(CO-like)	0.019251032	0.980748968
TRINITY_DN95472_c1_g2(COMT)	TRINITY_DN97293_c2_g7(CO-like)	0.023617737	0.976382263
TRINITY_DN92541_c0_g1(CAD)	TRINITY_DN97370_c2_g1(Nin-like)	0.038180563	0.961819437
TRINITY_DN87383_c2_g1(PAL)	TRINITY_DN97370_c2_g1(Nin-like)	0.027742063	0.972257937
TRINITY_DN97112_c1_g2(HCT)	TRINITY_DN97370_c2_g1(Nin-like)	0.017624784	0.982375216
TRINITY_DN95472_c1_g2(COMT)	TRINITY_DN97370_c2_g1(Nin-like)	0.013113411	0.986886589
TRINITY_DN83539_c2_g6(C4H)	TRINITY_DN97370_c2_g1(Nin-like)	0.014021466	0.985978534
TRINITY_DN87383_c2_g1(PAL)	TRINITY_DN97533_c2_g5(MYB)	0.033884668	0.966115332
TRINITY_DN97112_c1_g2(HCT)	TRINITY_DN97533_c2_g5(MYB)	0.028261101	0.971738899
TRINITY_DN95472_c1_g2(COMT)	TRINITY_DN97533_c2_g5(MYB)	0.023289063	0.976710937
TRINITY_DN83539_c2_g6(C4H)	TRINITY_DN97533_c2_g5(MYB)	0.001899618	0.998100382
TRINITY_DN92541_c0_g1(CAD)	TRINITY_DN97539_c1_g1(TALE)	0.019749953	0.980250047
TRINITY_DN87383_c2_g1(PAL)	TRINITY_DN97539_c1_g1(TALE)	0.023840051	0.976159949
TRINITY_DN97112_c1_g2(HCT)	TRINITY_DN97539_c1_g1(TALE)	0.026864237	0.973135763
TRINITY_DN95472_c1_g2(COMT)	TRINITY_DN97539_c1_g1(TALE)	0.032529256	0.967470744

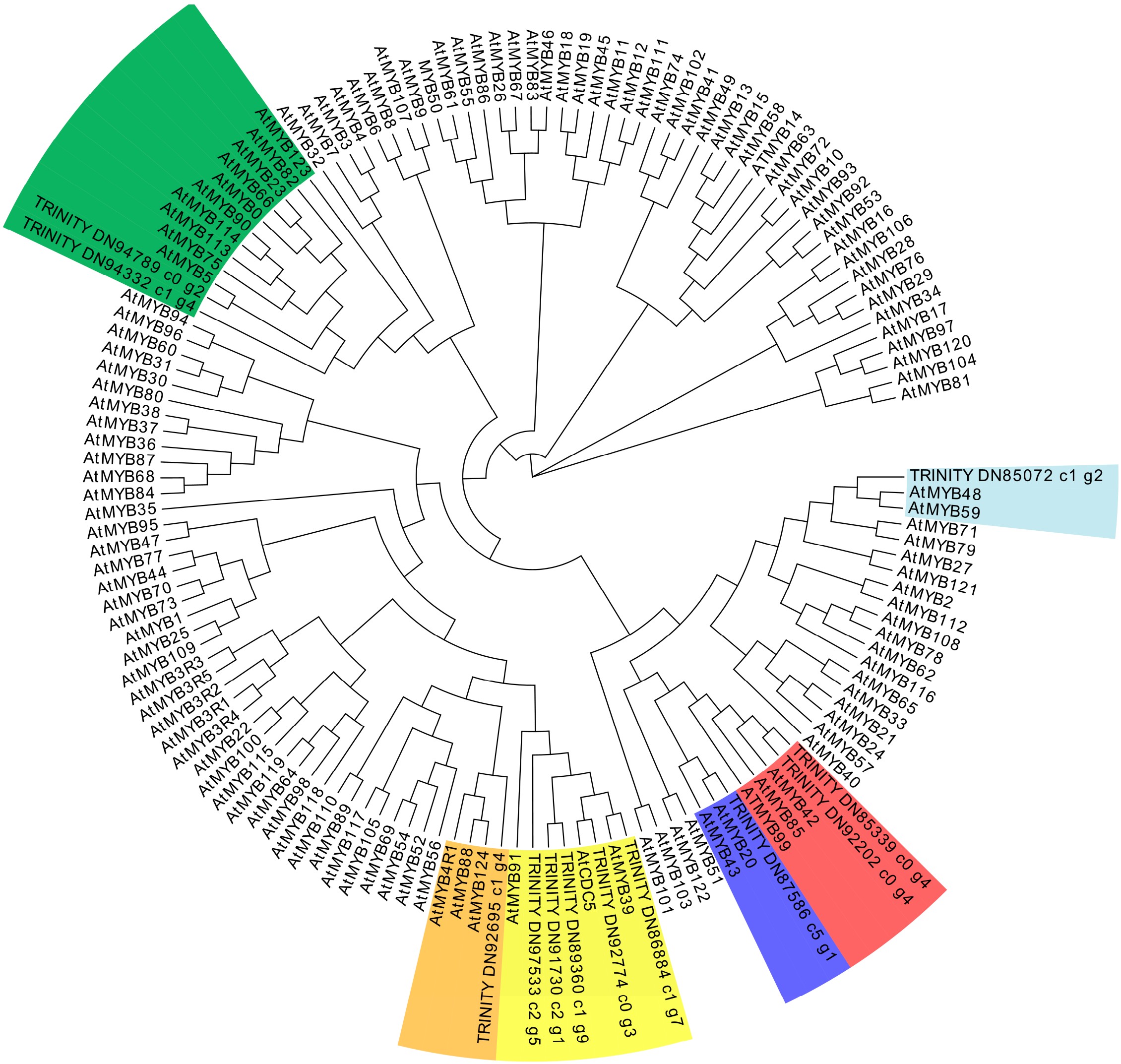

Supplementary material 3. Phylogenetic analysis of ten *Cyclocarya paliurus* MYB transcription factors and *Arabidopsis thaliana* MYB transcription factor genes. Different color boxes represent the clustering results of different transcription factors.

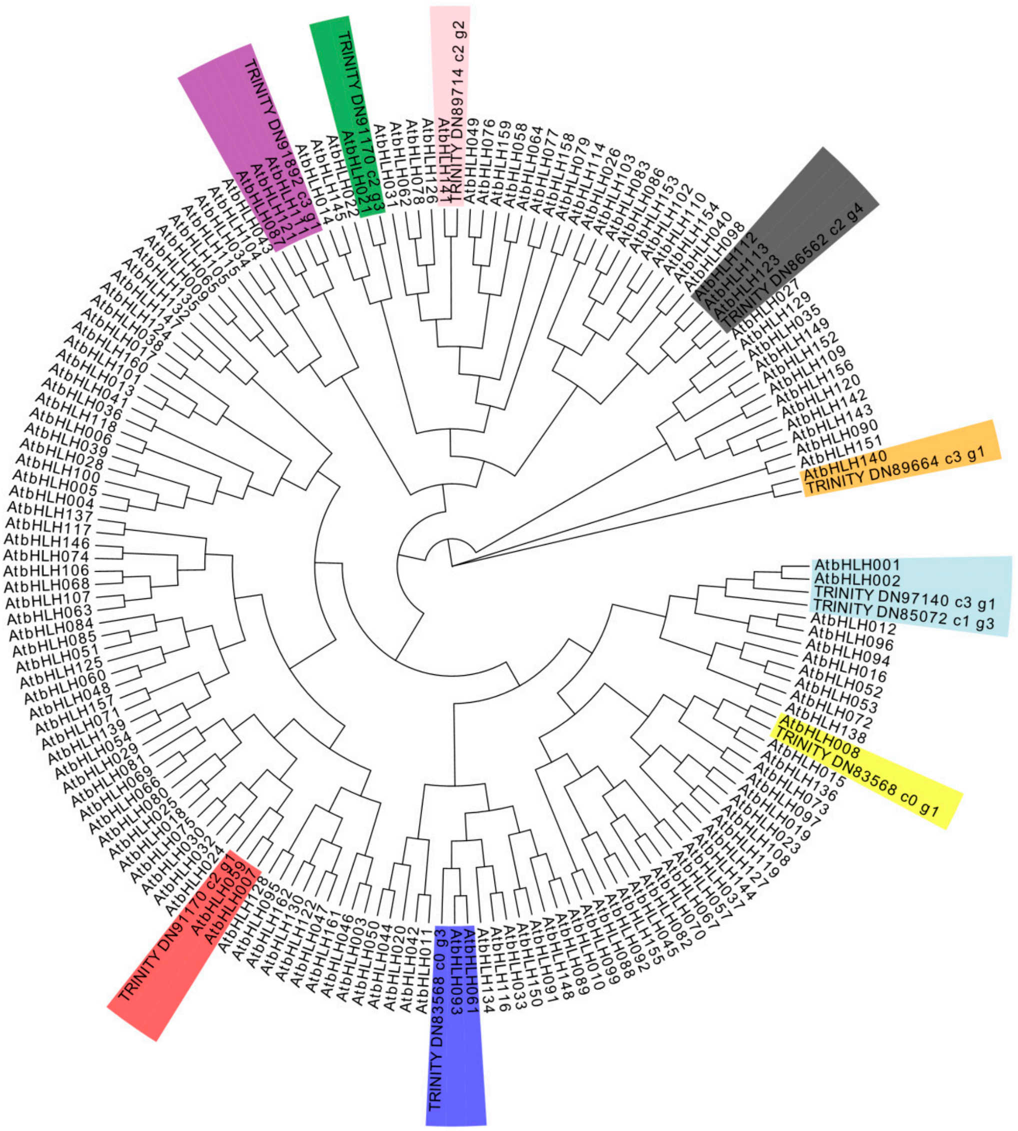

Supplementary material 4. Phylogenetic analysis of ten *Cyclocarya paliurus* bHLH transcription factors and *Arabidopsis thaliana* bHLH transcription factor genes. Different color boxes represent the clustering results of different transcription factors.

Supplementary material 5 qRT-PCR primers used in the study

No.	GENE ID	Sense primer	Anti-sense primer
1	TRINITY_DN87383_c2_g1	5'-CACAGGCGAACCAAACAA-3'	5'-GCAATGTAGGCAAGAGGG-3'
2	TRINITY_DN83539_c2_g6	5'-AACTTGATACGGTGCTTG-3'	5'-GTCGAAACTCTTCTGGGT-3'
3	TRINITY_DN95472_c1_g2	5'-GACAACGGGAAGGTGATT-3'	5'-ACTCGGAACCCTTGAAAT-3'
4	TRINITY_DN92541_c0_g1	5'-GAGCACCAACGCCGAACA-3'	5'-CCCGCAAACATCCATCATCT-3'
5	TRINITY_DN93270_c1_g1	5'-TGAGCAACCATCGGAGTC-3'	5'-CCGTCGGGCAAGTAATCT-3'
6	TRINITY_DN97112_c1_g2	5'-CGAACCACTTCTGCCTCA-3'	5'-CACCACCATTTCCATGTCTT-3'
7	TRINITY_DN85072_c1_g2	5'-GGTGGGAGACAAATAATAGG-3'	5'-TGAAGTTCGAGCACAAGG-3'
8	TRINITY_DN85339_c0_g4	5'-CTCGTCTTGGAAATAGGTGGTC-3'	5'-GCGTGACAGAGGGCTTGA-3'
9	TRINITY_DN86884_c1_g7	5'-CGGATTCCCGATGCCTAA-3'	5'-GTCCGTGTTGGTATTCTGGTTC-3'
10	TRINITY_DN87586_c5_g1	5'-CTGAGGAGGACAAGAAGC-3'	5'-GTGGGTGTTCCAGTGATT-3'
11	TRINITY_DN89360_c1_g9	5'-CTTCAATGGTTCCCAGCAA-3'	5'-ACTTCGACCCTCCCTTCC-3'
12	TRINITY_DN91730_c2_g1	5'-CATTGGGAGAAGATTGCT-3'	5'-CCTTCCTCTATTGCTCGT-3'
13	TRINITY_DN92202_c0_g4	5'-AGAGTTGCCGTCTTCGTT-3'	5'-AATCTTGGACCACCTGTT-3'
14	TRINITY_DN92695_c1_g4	5'-ACCCTGGTCCGTTGAGTT-3'	5'-ACATTTCCTGCTGTGCTT-3'
15	TRINITY_DN92774_c0_g3	5'-ATTACTGCCACAACGACTG-3'	5'-GACCCAACAAATGCTTCA-3'
16	TRINITY_DN94332_c1_g4	5'-TCATTATCCGATTACATTCC-3'	5'-TCTTGCTGCTGATCTTTG-3'
17	TRINITY_DN94789_c0_g2	5'-CAGGCTAAGATGGTTGAA-3'	5'-TATCTCATTATCGGTTCG-3'
18	TRINITY_DN97533_c2_g5	5'-CCAGCAGCCAAGACAGAC-3'	5'-TCCGACGATGACAGAGTT-3'
19	β-Actin	5'-CTCTTCCAGCCATCCATGATCG-3'	5'-CCACTGAGGACAATATTGCCAT-3'
